# *Fusarium*-Derived Secondary Metabolites with Antimicrobial Effects

**DOI:** 10.3390/molecules28083424

**Published:** 2023-04-13

**Authors:** Meijie Xu, Ziwei Huang, Wangjie Zhu, Yuanyuan Liu, Xuelian Bai, Huawei Zhang

**Affiliations:** 1 School of Pharmaceutical Sciences, Zhejiang University of Technology, Hangzhou 310014, China; 2 College of Life and Environmental Sciences, Hangzhou Normal University, Hangzhou 311121, China

**Keywords:** *Fusarium*, secondary metabolite, antimicrobial effect, antibacterial, antifungal, antiviral, antiparasitic

## Abstract

Fungal microbes are important in the creation of new drugs, given their unique genetic and metabolic diversity. As one of the most commonly found fungi in nature, *Fusarium* spp. has been well regarded as a prolific source of secondary metabolites (SMs) with diverse chemical structures and a broad spectrum of biological properties. However, little information is available concerning their derived SMs with antimicrobial effects. By extensive literature search and data analysis, as many as 185 antimicrobial natural products as SMs had been discovered from *Fusarium* strains by the end of 2022. This review first provides a comprehensive analysis of these substances in terms of various antimicrobial effects, including antibacterial, antifungal, antiviral, and antiparasitic. Future prospects for the efficient discovery of new bioactive SMs from *Fusarium* strains are also proposed.

## 1. Introduction 

Antimicrobial agents play a significant role in the treatment of infectious diseases caused by pathogenic microorganisms with various modes of action. Since the fortuitous discovery of penicillin in 1928, hundreds of antibiotics have been approved for clinical use. However, some of these drugs have become less efficacy or unavailability simultaneously owing to the development of antimicrobial resistance (AMR), in which a pathogenic microbe evolves a survival mechanism that protects the drug target by modification or replacement, or degradation or modification of the antibiotic to render it harmless, such as MRSA (methicillin-resistant *Staphylococcus aureus*), multidrug-resistant *S. aureus* (MDRS), VREF (vancomycin-resistant *Enterococcus faecium*), CRKP (cephalosporin-resistant *Klebsiella pneumoniae*) [1]. Antimicrobial resistance has become an increasing threat to human health and is widely considered to be the next global pandemic [2]. Therefore, it is an urgent need for the discovery of new antimicrobial drugs with novel structural scaffolds and new modes of action.

Microorganisms are well recognized as a prolific source of biomolecules with diverse chemical structures and various biological properties. Microbial natural products have been, to date, our most successful defense against infectious disease. As one of the most commonly isolated filamentous fungi in terrestrial and marine environments, *Fusarium* spp. possess the potential capability to biosynthesize structurally diverse secondary metabolites (SMs), including alkaloids, peptides, amides, terpenoids, quinones, pyranones, and miscellaneous compounds [3]. Up to now, however, no document highlighting *Fusarium*-derived SMs with antimicrobial effects has been reported. With the aim to enrich our knowledge, this review comprehensively summarizes the occurrence of these antimicrobial substances, including antibacterials, antifungals, antivirals, and antiparasitics.

As of December 2022, the Dictionary of Natural Products (DNP) database listed 783 *Fusarium*-derived SMs, many of them also occurring in other microbial genera. By extensive literature search, as many as 185 antimicrobial SMs (**1**–**185**) had been discovered from *Fusarium* strains and are, respectively, introduced in terms of various antimicrobial activities, including antibacterial, antifungal, antiviral, and antiparasitic. Their detailed information is supplied in the Supplementary Materials.

## 2. Antibacterial Secondary Metabolites

**Figure 1:**
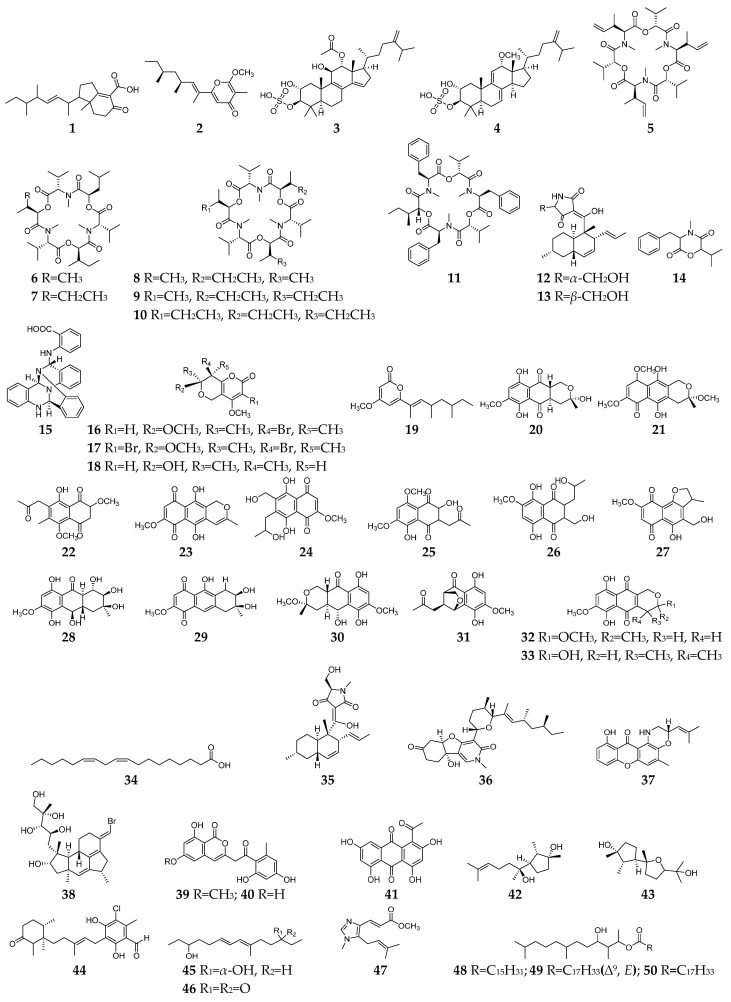
*Fusarium*-derived anti-Gram-positive bacterial SMs (**1**–**50**).

**Figure 2:**
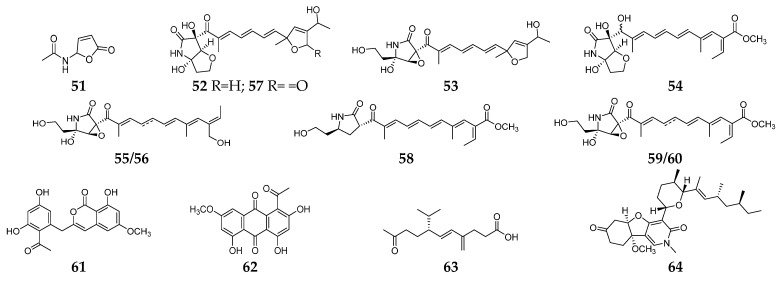
*Fusarium*-derived anti-Gram-negative bacterial SMs (**51**–**64**).

**Figure 3:**
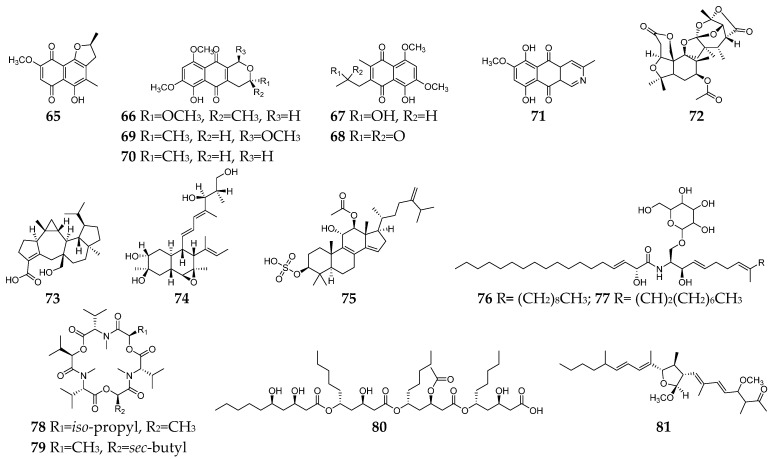
*Fusarium*-derived anti-Gram-positive and anti-Gram-negative bacterial SMs (**65**–**81**).

Bacterial infection is a common clinical disease that can affect a variety of organs and tissues. *Fusarium*-derived antibacterial SMs have a wide array of structural motifs, most of which are polyketides, followed by alkaloids, terpenoids, and cyclopeptides. According to antibacterial properties, these chemicals are divided into three groups, including anti-Gram-positive bacterial SMs (**1**–**50**, Figure 1), anti-Gram-negative bacterial SMs (**51**–**64**, Figure 2) and both anti-Gram-positive and anti-Gram-negative bacterial SMs (**65**–**81**, Figure 3).

### 2.1. Anti-Gram-Positive Bacterial SMs

Fifty *Fusarium*-derived SMs (**1**–**50**, Figure 1) had been characterized and displayed various bactericidal effects on Gram-positive strains, such as *Staphylococcus aureus*, methicillin-resistant *Staphylococcus aureus*, multidrug-resistant *S. aureus*, *Mycobacterium tuberculosis*, *Bacillus subtilis*, etc. Fusariumins C (**1**) and D (**2**) are two new polyketides produced by an endophytic strain *F. oxysporum* ZZP-R1 from coastal plant *Rumex midair* Makino displayed medium effect on *S. aureus* with MIC (minimum inhibitory concentration) values of 6.25 and 25.0 μM, respectively [4]. Two triterpene sulfates (**3** and **4**) isolated from *F. compactum* exhibited weak activity toward *S. aureus* and *Streptococcus* strains in the range of 6–50 µg/mL [5]. Enniatins (**5**–**10**), a group of antibiotics commonly synthesized by various *Fusarium* strains, are six-membered cyclic depsipeptides formed by the union of three molecules of D-α-hydroxyisovaleric acid and three *N*-methyl-*L*-amino acids [6]. Three enniatins (**8**–**10**), beauvericin A (**11**) and trichosetin (**12**) were obtained from an endophytic fungus, *Fusarium* sp. TP-G1 and showed moderate anti-*S. aureus* and anti-methicillin-resistant *S. aureus* effects with MIC values in the range of 2–16 µg/mL [7]. Two enantiomers (**12** and **13**) were separated from the culture broth of *F. oxysporum* FKI-4553 and found to have an inhibitory effect on the undecaprenyl pyrophosphate synthase activity of *S. aureus* with IC_50_ values of 83 and 30 µM, respectively [8].

Lateritin (**14**) derived from *Fusarium* sp. 2TnP1–2 showed anti-*S. aureus* activity at 2 µg per disc with 7 mm of inhibition zone [9]. A new polycyclic quinazoline alkaloid (**15**) displayed moderate antibacterial activity against methicillin-resistant *S. aureus* and multidrug-resistant *S. aureus*, with the same MIC value of 6.25 µg/mL [10]. Three pyranopyranones (**16**–**18**) showed weak inhibitory activities against *S. aureus*, methicillin-resistant *S. aureus*, and multidrug-resistant *S. aureus* [11]. Compound **19** was a new pyran-2-one with weak activity against methicillin-resistant *S. aureus* and was shown to be the inhibitor of the quorum-sensing mechanism of *S. aureus* and *Pseudomonas aeruginosa* [12]. Trans-dihydrofusarubin (**20**) and seven analogs (**21**–**27**) had significant antibiotic activity against *S. aureus* (MIC values < 4 µg/mL), and compounds **26** and **27** exhibited potent activity against *S. pyogenes* [13]. Five naphthoquinones **28**–**32** showed anti-*Mycobacterium tuberculosis* activity with MICs ranging from 25 to 50 µg/mL [14]. Compounds **32** and **33** displayed moderate antibacterial activity against *S. aureus* and potent activities against *B. cereus* and *S. pyogenes* with MIC values of <1 µg/mL as compared to ciprofloxacin, whose MIC value was 0.15 and 10 µg/mL, respectively [15].

Linoleic acid (**34**) and *epi*-equisetin (**35**) had certain inhibitory activity against *S. aureus* and multidrug-resistant *S. aureus* [16]. (−)-4,6′-anhydrooxysporidinone (**36**) was obtained from *F. oxysporum* and showed weak anti-multidrug-resistant *S. aureus* and moderate anti-*B. subtilis* effects [17]. Fusaroxazin (**37**), a novel antimicrobial xanthone derivative from *F. oxysporum*, possessed significant antibacterial activity towards *S. aureus* and *B. cereus*, with MIC values of 5.3 and 3.7 µg/mL, respectively [18]. Neomangicol B (**38**) isolated from the mycelial extract of a marine *Fusarium* strain was found to inhibit *B. subtilis* growth with a potency similar to that of the antibiotic gentamycin [19]. Three aromatic polyketides (**39**–**41**) were produced by strain *F. proliferatum* ZS07 and possessed potent antibacterial activity against *B. subtilis* with the same MIC values of 6.25 µg/mL [20]. Two sesterterpenes (**42** and **43**) produced by *F. avenaceum* SF-1502 displayed stronger antibacterial activity against *B. megaterium* than positive controls (ampicillin, erythromycin, and streptomycin) [21]. 4,5-Dihydroascochlorin (**44**) had strong antibacterial activity towards *Bacillus megaterium* [22]. Fusariumnols A (**45**) and B (**46**) were two novel anti-*S. epidermidis* aliphatic unsaturated alcohols isolated from *F*. *proliferatum* 13,294 [23]. Fungerin (**47**) displayed weak antibacterial activity against *S. aureus* and *S. pneumoniae* [24]. Compounds **48**–**50** were purified from *F*. *oxysporum* YP9B and showed a potent inhibitory effect on *S. aureus*, *E.faecalis*, *S. mutans*, *B. cereus*, and *M. smegmatis* with MICs of less than 4.5 µg/mL [25].

### 2.2. Anti-Gram-Negative Bacterial SMs

Butenolide (**51**) was a fusarium mycotoxin from unknown origin strain *Fusaium* sp. and showed selective inhibitory activity against *E. coli* [26]. Extensive chemical investigation of the endophytic fungus *F. solani* JK10 afforded nine 2-pyrrolidone derivatives (**52**–**60**), which displayed antibacterial activity against *E. coli* with MIC values of 5–10 µg/mL. Particularly, three lucilactaene analogs (**52**–**54**) had strong inhibitory effects on *Acinetobacter* sp., comparable to the positive control streptomycin [27]. One new aromatic polyketide, karimunones B (**61**), together with compounds **62** and **63,** was obtained from sponge-associated *Fusarium* sp. KJMT.FP.4.3 and exhibited anti-multidrug resistant *Salmonella enterica* ser. Typhi activity with a MIC of 125 µg/mL [28]. Fusapyridon A (**64**) is produced by an endophytic strain, *Fusarium* sp. YG-45 demonstrated moderate antibacterial activity against *Pseudomonas aeruginosa* with a MIC value of 6.25 µg/mL [29].

### 2.3. Both Anti-Gram-Positive and Anti-Gram-Negative Bacterial SMs

Seventeen *Fusarium*-derived SMs (**65**–**81**, Figure 3) were shown to have both anti-Gram-positive and anti-Gram-negative activity. Seven naphthoquinones (**65**–**71**) demonstrated moderate activities against an array of Gram-positive and Gram-negative bacteria, such as *B. megaterium*, *B. subtilis*, *C. perfringens*, *E. coli*, methicillin-resistant *S. aureus*, *P. aeruginosa*, *S. aureus*, and *S. pyogenes* [13,21,30,31]. The mechanism of action (MoA) study indicated that compounds **66** and **71** could stimulate the oxygen consumption of bacterial cells and induce cyanide-insensitive oxygen consumption, which results in the generation of superoxide anion and hydrogen peroxide [32]. Compounds **72**–**75** were polycyclic terpenoids, respectively, produced by three *Fusarium* strains [33,34,35]. Compound **72** had significant activity against *S. aureus* and *P. aeruginosa* with a MIC value of 6.3 µg/mL, and **73** showed moderate activities against *Salmonella enteritidis* and *Micrococcus luteus* with MIC values of 6.3 and 25.2 µg/mL, respectively, while **74** showed a broad spectrum of antibacterial activity and **75** exhibited moderate antibacterial activities against *S. aureus* and *E. coli* with the same MIC value of 16 µg/mL. Two xanthine oxidase inhibitory cerebrosides (**76** and **77**) were identified and purified from the culture broth of *Fusarium* sp. IFB-121 and showed strong antibacterial activities against *B. subtilis*, *E. coli*, and *P. fluorescens* with MICs of less than 7.8 µg/mL [36]. Enniatins J_1_ (**78**) and J_3_ (**79**) were two hexadepsipeptides with an array of antibacterial activity toward *C. perfringens*, *E. faecium*, *E. coli*, *S. dysenteriae*, *S. aureus*, *Y. enterocolitica*, and lactic acid bacteria except for *B. adolescentis* [37]. Halymecin A (**80**) was produced by a marine-derived *Fusarium* sp. FE-71-1 and exhibited a moderate inhibitory effect on *E. faecium*, *K. pneumoniae*, and *P. vulgaris* with the MIC value of 10 µg/mL [38]. Fusaequisin A (**81**) was isolated from rice cultures of *F. equiseti* SF-3-17 and found to have moderate antimicrobial activity against *S. aureus* NBRC 13,276 and *P. aeruginosa* ATCC 15,442 [39].

## 3. Antifungal Secondary Metabolites

**Figure 4:**
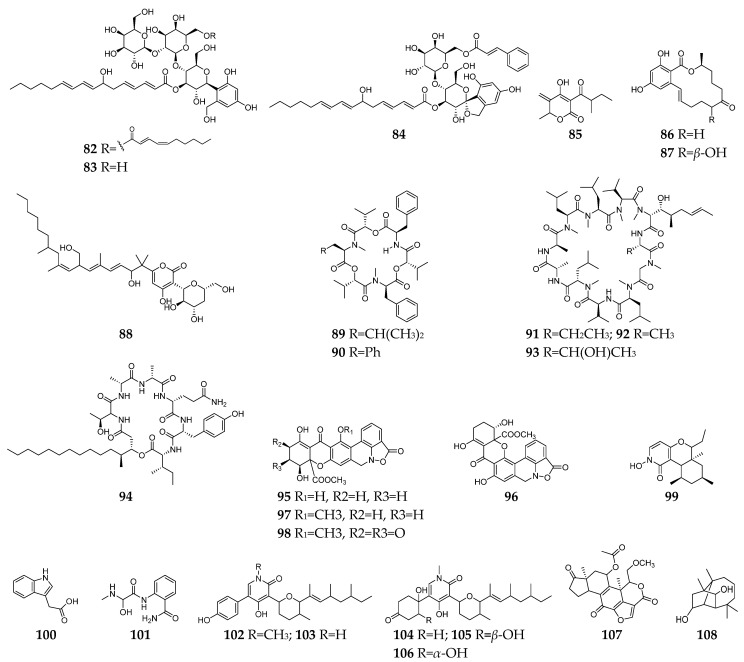
*Fusarium*-derived antifungal SMs (**82**–**108**).

Invasive fungal infections are very common in immunocompromised patients (such as acquired immune deficiency syndrome and organ transplantation) and have become a global problem resulting in 1.7 million deaths every year [40,41,42]. Furthermore, the overuse of antifungal agents increases opportunistic pathogen resistance, which had been listed as one of the dominant threats by the World Health Organization in 2019. Therefore, the urgent need for new antimycotics with novel targets is undeniable. Till the end of 2022, twenty-seven antifungal SMs (**82**–**108**, Figure 4) had been discovered from *Fusarium* strains. Compounds **82**–**84** are three anti-*C. albicans* glycosides belong to the papulacandin class [43,44]. The MoA study suggested that compound **82** is an inhibitor of glutamine synthetase (GS) enzyme for (l,3)-*β*-glucan biosynthesis [43]. CR377 (**85**) was a new α-furanone derivative from an endophytic *Fusarium* sp. CR377 and showed a similar antifungal effect on *C. albicans* with nystatin [45]. Compounds **86** and **87** were two zearalenone analogs and exhibited weak activity against *Cryptococcus neoformans* [46]. Neofusapyrone (**88**) produced by a marine-derived *Fusarium* sp. FH-146 displayed moderate activity against *A. clavatus* F318a with a MIC value of 6.25 µg/mL [47]. Six cyclic depsipeptides **89**–**94** had been isolated from several *Fusarium* strains and found to have significant inhibitory activities against pathogenic fungi, such as *C. albicans* [48], *C. glabrata*, *C. krusei*, *V. ceratosperma*, and *A. fumigates* [49]. Cyclosporin A (**91**) has long been recognized as an immunosuppressant agent and could inhibit the growth of sensitive fungi after their germination [50,51]. Parnafungins A-D (**95**–**98**) were isoxazolidinone-containing natural products and demonstrated broad-spectrum antifungal activity with no observed activity against bacteria. The targeted pathway of these alkaloids was determined to be the mRNA 3`-cleavage and polyadenylation process [52,53]. One *N*-hydroxypyridine derivative (**99**) showed antifungal activity against *C. albicans* and *Penicillium chrysogenum* with MICs of 16 and 8 µg/mL, respectively [54]. Indole acetic acid (**100**) exhibited activity against the fluconazole-resistant *C. albicans* (MIC = 125 µg/mL) [55].

Fusaribenzamide A (**101**) possessed a significant anti-*C. albicans* activity with MIC of 11.9 µg/disc compared to nystatin (MIC = 4.9 µg/disc) [56]. Three pyridone derivatives (**102**–**104**) displayed significant activities against multidrug-sensitive *S. cerevisiae* 12geneΔ0HSR-iERG6, and the MoA study indicated that these substances have a potent inhibitory effect on NADH-cytochrome C oxidoreductase [57]. Compounds **105**–**107** were derived from strain *F. oxysporum* N17B, and the former (**105** and **106**) showed selective fungistatic activity against *Aspergillus fumigatus*, and the latter (**107**) had selective potent activity against *C. albicans* through inhibition of phosphatidylinositol 3-kinase [58]. Culmorin (**108**) displayed remarkable antifungal activity against both marine (*S. marina*, *M. pelagica*) and medically relevant fungi (*A. fumigatus*, *A. niger*, *C. albicans*, *T. mentagrophytes*) [59,60].

## 4. Both Antibacterial and Antifungal Secondary Metabolites

**Figure 5:**
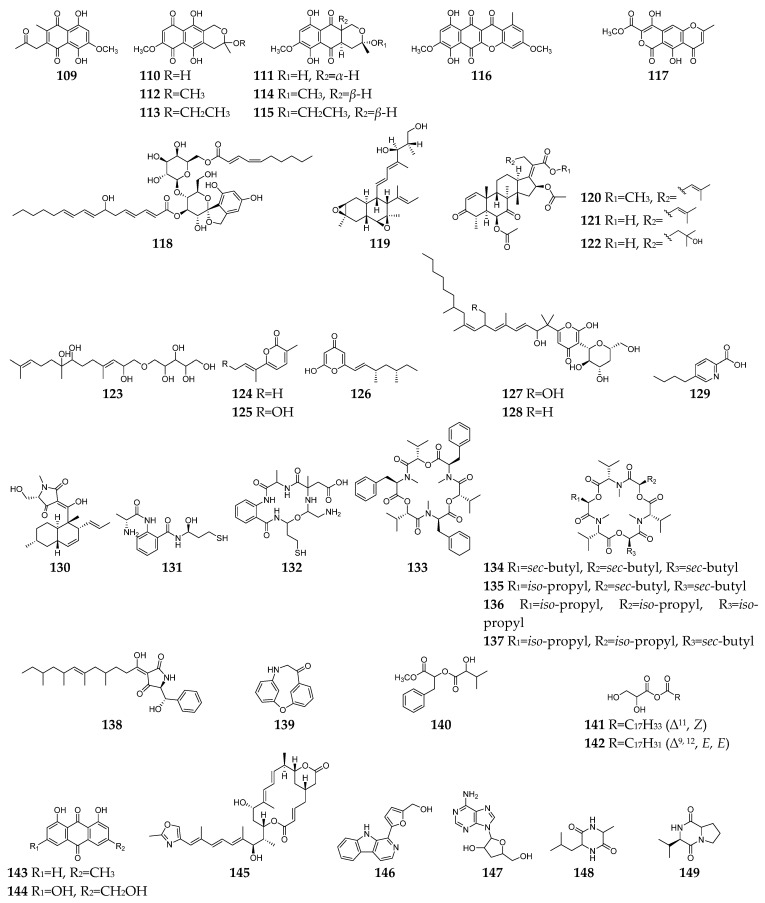
*Fusarium*-derived antibacterial and antifungal SMs (**109**–**149**).

Till the end of 2022, forty-one SMs (**109**–**149**, Figure 5) with both antibacterial and antifungal effects had been discovered from *Fusarium* spp. Among these *Fusarium*-derived 1,4-naphthoquinone analogs (**109**–**115**), compound **109** showed potent anti-Gram-positive bacteria activity against *B. cereus* and *S. pyogenes* with MIC of <1 µg/mL and anti-*C. albicans* activity with IC_50_ (the half maximal inhibitory concentration) of 6.16 µg/mL [14], and **110**–**115** demonstrated moderate inhibitory effects on *S. aureus*, *C. albicans*, and *B. subtilis* [61]. Bikaverin (**116**) was found to have anti-*E. coli* and antifungal (*P. notatum*, *Alternaria humicola*, and *A. flavus*) activity [48,62,63]. Lateropyrone (**117**) was the same SM as *F. acuminatum*, *F. lateritium*, and *F. tricinctum* and displayed good antibacterial activity against *B. subtilis*, *S. aureus*, *S. pneumoniae*, methicillin-resistant *S. aureus*, *Mycobacterium tuberculosis*, and vancomycin-resistant of *E. faecalis* and significant inhibitory activity towards the growth of *C. albicans* [64,65,66,67]. BE-29,602 (**118**) was a novel antibiotic of the papulacandin family, showing good activity against *C. albicans*, *S. cerevisiae*, *S. pombe* with MIC values < 1 µg/mL and moderate activity against *B. subtilis* and *P. chrysogenum* with the MIC values < 8 µg/mL [44,68]. Fusarielin A (**119**) was a meroterpenoid with moderate antifungal activities against *A. fumigatus* and *F. nivale* and weak antibacterial effect on *S. aureus*, methicillin-resistant *S. aureus*, and multidrug-resistant *S. aureus* [11,69]. Three helvolic acid derivatives (**120**–**122**) displayed potent antifungal and antibacterial activities against *B. subtilis*, *S. aureus*, *E. coli*, *B. cinerea*, *F. Graminearum*, and *P. capsica* [70]. Fusartricin (**123**) had moderate antimicrobial activity against *E. aerogenes*, *M. tetragenu*, and *C. albicans* with the same MIC value of 19 µM [34].

Compounds **124**–**128** are pyrone family members and showed antimicrobial activity against bacteria (such as *B. subtilis*, *S. aureus*, *Vibrio parahaemolyticus*, *C. kefyr*, and *P. aeruginosa*) and fungi (such as *A. clavatus*, *Geotrichum candidum*, *C. albicans*, *M. albican*, and *S. cerevisiae*) [47,71,72,73,74]. Fusaric acid (**129**), one of the most significant mycotoxins from *Fusarium* strains, displayed a broad spectrum of moderate antimicrobial activity against *Bacillus* species, *Acinetobacter baumannii, Phytophthora infestans*, etc. [75,76,77]. Equisetin (**130**) was shown to be active against several strains of Gram-positive bacteria (*B. subtilis*, *Mycobacterium phlei*, *S. aureus*, *methicillin-resistant S. aureus*, and *S. erythraea*) and the Gram-negative bacteria *Neisseria perflava* at concentrations of 0.5–4.0 µg/mL, as well as antifungal activity toward *P. syringae* and *R. cerealis* [78,79]. Fusarithioamides A (**131**) and B (**132**) demonstrated antibacterial potential towards *B. cereus*, *S. aureus*, and *E. coli* compared to ciprofloxacin and selective antifungal activity towards *C. albicans* compared to clotrimazole [80,81]. Beauvericin (**133**) and enniatins A, A1, B and B1 (**134**–**137**) are cyclic hexadepsipeptides with a wide array of highly antimicrobial activities against bacteria (such as *B. subtilis*, *S. aureus*, *methicillin-resistant S. aureus*, etc.) and fungi (such as *C. albicans*, *B. bassiana*, *T. harzianum*, etc.) [82,83,84,85,86]. Unlike most antibiotics, cell organelles or enzyme systems are the targets of the antibiotic **133** [87]. As a drug efflux pump modulator, furthermore, compound **133** had the capability to reverse the multi-drug resistant phenotype of *C. albicans* by blocking the ATP-binding cassette transporters and to repress the expression of many filament-specific genes, including the transcription factor BRG1, global regulator TORC1 kinase [88]. Fusaramin (**138**) displayed anti-Gram-positive and anti-Gram-negative bacterial activity and could inhibit the growth of *S. cerevisiae* 12geneΔ0HSR-iERG6 [57]. Compounds **139**–**142** were isolated from *F. oxysporum* YP9B and exhibited a significant antimicrobial effect against bacterial and fungi at concentrations of 0.8–6.3 µg/mL [25]. Seven SMs (**143**–**149**) were separated from an endophytic fungus *F. equiseti*, and showed antibacterial (such as *B. subtilis*, *S. aureus*, *B. megaterium*) and anti-*C. albicans* activities [89].

## 5. Antiviral Secondary Metabolites

**Figure 6:**
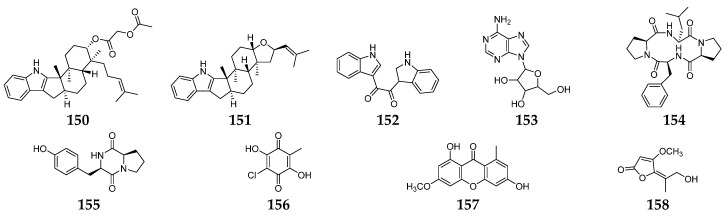
*Fusarium*-derived antiviral SMs (**150**–**158**).

The infections by viruses in humans resulted in millions of deaths globally and are accountable for viral diseases, including HIV/AIDS, hepatitis, influenza, herpes simplex, common cold, etc. [90]. The emergence of new viruses like Ebola and coronaviruses (SARS-CoV, SARS-CoV-2) emphasizes the need for more innovative strategies to develop better antiviral drugs. Twenty-three *Fusarium*-derived SMs (**64**, **99**, **105**, **135**–**137**, **140**–**142**, **144**–**147**, **149**–**158**, Figure 6) had been shown to have antiviral effects. The isolation of fusaricide (**99**) was guided by the Rev (regulation of virion expression) binding assay [54]. Fusapyridon A (**64**) and oxysporidinone (**105**) displayed antiviral activity against the coronavirus (HCoV-OC43) with IC_50_ values of 13.33 and 6.65 μM, respectively [91]. Their enniatins (**135**–**137**) were found to protect human lymphoblastoid cells from HIV-1 infection with an in vitro “therapeutic index” of approximately 200 (IC_50_ = 1.9, EC_50_ = 0.01 µg/ mL, respectively) [92]. The antiviral activity against HSV type-1 was determined to be 0.312 µM for compound **140** and 1.25 µM for **141** and **142** [25]. Three indole alkaloids (**150**–**152**) were obtained from a marine-derived *Fusarium* sp. L1 and exhibited inhibitory activity against the Zika virus (ZIKV) with EC_50_ values of 7.5, 4.2, and 5.0 μM, respectively [93]. A chemical study of an endophytic fungus *F. equiseti* led to the isolation of compounds **144**–**147** and **153**–**157**, of which **149** and **157** showed good potency against hepatitis C virus NS3/4A protease, while **144** and **155** were the most potent hepatitis C virus NS3/4A protease inhibitors [89]. Coculnol (**158**) was a penicillic acid from a coculture of *F. solani* FKI-6853 and *Talaromyces* sp. FKA-65 displayed an inhibitory effect on A/PR/8/34 (H1N1) with an IC_50_ value of 283 µg/mL [94].

## 6. Antiparasitic Secondary Metabolites

**Figure 7:**
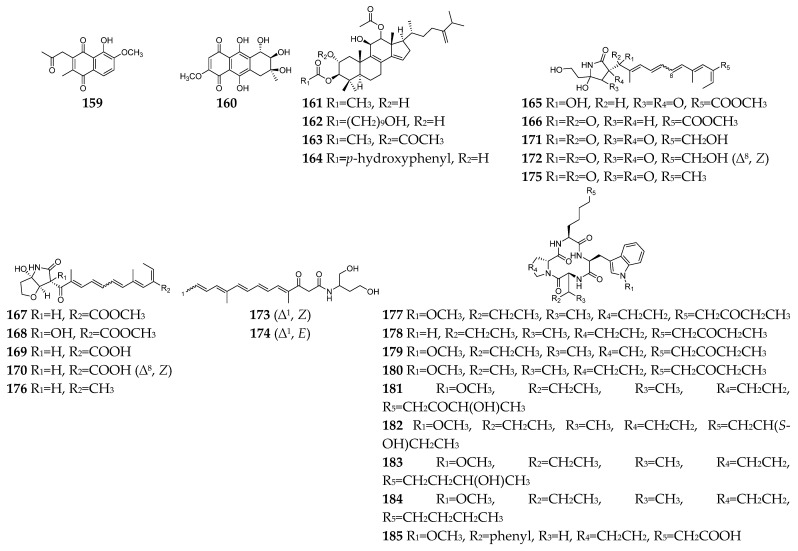
*Fusarium*-derived antiparasitic SMs (**159**–**185**).

Parasitic diseases caused by protozoa, helminths and ectoparasites affect millions of people each year and result in substantial morbidity and mortality, particularly in tropical regions [95]. Therefore, new antiparasitic agents are urgently needed to treat and control these diseases. A total of 39 antiparasitic SMs (**23**, **28**, **29**, **59**, **108**, **93**, **116**, **133**–**137**, **159**–**185**, Figure 7) had been isolated and characterized from *Fusarium* strains. Five naphthoquinones (**23**, **29**, **30**, **109,** and **159**) and one anthraquinone (**160**) showed weak inhibitory activity toward the most deadly malaria parasite *Plasmodium falciparum* K1 with IC_50_ values in the range 9.8–26.1 µM [96]. However, compound **93** displayed significant antiplasmodial activity toward *P. falciparum* (D6 clone) with an IC_50_ value of 0.34 µM [49]. Bikaverin (**116**) was specifically effective against *Leishmania brasiliensis*, which is one of the main causes of cutaneous leishmaniasis in the Americas [97]. Beauvericin (**133**) was reported to inhibit *Trypanosoma cruzi* with an IC_50_ value of 2.43 μM and *L. braziliensis* with an EC_50_ value of 1.86 μM [98,99]. In addition to antibacterial and antifungal effects, enniatins (**134**–**137**) exhibited mild anti-leishmanial activity by inhibition of the activity of thioredoxin reductase enzyme of *P. falciparum* [6]. Integracides F, G, H, and J (**161**–**164**) were also shown to have stronger anti-leishmanial activity towards *L. donovani* than the positive control pentamidine (IC_50_ = 6.35 µM) [100]. Among twelve lucilactaene derivatives (**165**–**176**), compounds **166**–**168** showed very potent antimalarial activity toward *P. falciparum* (IC_50_ = 0.0015, 0.15, and 0.68 μM, respectively) [101,102,103]. Structure−activity relationship study suggested that epoxide is extremely detrimental, and demethylation of the lucilactaene methyl ester and formation of the free carboxylic acid group resulted in a 300-fold decrease in activity. Nine cyclic tetrapeptides (**177**–**185**) are apicomplexan histone deacetylase (HDA) inhibitors [104,105,106]. Particularly, compound **177** was an excellent inhibitory agent (IC_50_ < 2 nM) and showed in vivo high efficacy against *P. berghei* malaria in mice at less than 10 mg/kg.

## 7. Conclusions

In summary, the genus *Fusarium* is one of the excellent producers of antimicrobial SMs, some of which have great potential in new drug development, such as anti-Gram-positive bacterial terpenes **38**, **42** and **43**, anti-Gram-negative lucilactaenes **52**–**54**, antifungal papulacandin **82** and pyridones **102**–**104**, antiviral enniatins **135**–**137**, and antiparasitic integracides **161**–**164**, etc. In the past two decades, however, the rate of discovery of novel SMs from *Fusarium* has constantly been decreasing [3]. Fortunately, a growing number of evidence suggest that the potential of *Fusarium* spp. to make novel SMs is still immense since most of their SM biosynthetic gene clusters (BGCs) are inactive or un-awakened under traditional fermentation and culture conditions [107]. More and more cryptic BGCs responsible for the biosynthesis of novel SMs have been disclosed by various bio-informative tools and approaches and efficiently activated using genome mining strategies, such as BGC heterogeneous expression [108], promoter engineering [109] and gene transcriptional regulation [110]. In addition, more efforts should be made to analyze and interpret the action mechanisms of *Fusarium*-derived leading compounds, which have similar or more potent antimicrobial effects compared to positive controls.

## Data Availability

The data presented in this study are available in the Supplementary Material.

## Supplementary Materials

The following supporting information can be downloaded at: https://www.mdpi.com/article/10.3390/molecules28083424/s1, Table S1: Detail information for *Fusarium*-derived anti-Gram-positive bacterial SMs; Table S2: Detail information for *Fusarium*-derived anti-Gram-negative bacterial SMs; Table S3: Detail information for *Fusarium*-derived both anti-Gram-positive and anti-Gram-negative bacterial SMs; Table S4: Detail information for *Fusarium*-derived antifungal SMs; Table S5: Detail information for *Fusarium*-derived both antibacterial and antifungal SMs; Table S6: Detail information for *Fusarium*-derived antiviral SMs; Table S7: Detail information for *Fusarium*-derived antiparasitic SMs.

## References

[1] Denissen J., Reyneke B., Waso-Reyneke M., Havenga B., Barnard T., Khan S., Khan W. (2022). Prevalence of ESKAPE pathogens in the environment: Antibiotic resistance status, community-acquired infection and risk to human health. Int. J. Hyg. Environ. Health.

[2] Nadimpalli M.L., Chan C.W., Doron S. (2021). Antibiotic resistance: A call to action to prevent the next epidemic of inequality. Nat. Med..

[3] Li M., Yu R., Bai X., Wang H., Zhang H. (2020). *Fusarium*: A treasure trove of bioactive secondary metabolites. Nat. Prod. Rep..

[4] Chen J., Bai X., Hua Y., Zhang H., Wang H. (2019). Fusariumins C and D, two novel antimicrobial agents from *Fusarium oxysporum* ZZP-R1 symbiotic on Rumex madaio Makino. Fitoterapia.

[5] Brill G.M., Kati W.M., Montgomery D., Karwowski J.P., Humphrey P.E., Jackson M., Clement J.J., Kadam S., Chen R.H., McAlpine J.B. (1996). Novel triterpene sulfates from *Fusarium compactum* using a rhinovirus 3C protease inhibitor screen. J. Antibiot..

[6] Zaher A.M., Makboul M.A., Moharram A.M., Tekwani B.L., Calderon A.I. (2015). A new enniatin antibiotic from the endophyte *Fusarium tricinctum* Corda. J. Antibiot..

[7] Shi S., Li Y., Ming Y., Li C., Li Z., Chen J., Luo M. (2018). Biological activity and chemical composition of the endophytic fungus *Fusarium* sp. TP-G1 obtained from the root of *Dendrobium officinale* Kimura et Migo. Rec. Nat. Prod..

[8] Inokoshi J., Shigeta N., Fukuda T., Uchida R., Nonaka K., Masuma R., Tomoda H. (2013). *Epi*-trichosetin, a new undecaprenyl pyrophosphate synthase inhibitor, produced by *Fusarium oxysporum* FKI-4553. J. Antibiot..

[9] Du Z., Song C., Yu B., Luo X. (2008). Secondary metabolites produced by *Fusarium* sp. 2TnP1-2, an endophytic fungus from *Trewia nudiflora*. Chin. J. Med. Chem..

[10] Nenkep V., Yun K., Son B.W. (2016). Oxysporizoline, an antibacterial polycyclic quinazoline alkaloid from the marine-mudflat-derived fungus *Fusarium oxysporum*. J. Antibiot..

[11] Nenkep V., Yun K., Zhang D., Choi H.D., Kang J.S., Son B.W. (2010). Induced production of bromomethylchlamydosporols A and B from the marine-derived fungus *Fusarium tricinctum*. J. Nat. Prod..

[12] Alfattani A., Marcourt L., Hofstetter V., Queiroz E.F., Leoni S., Allard P.M., Gindro K., Stien D., Perron K., Wolfender J.L. (2021). Combination of pseudo-LC-NMR and HRMS/MS-based molecular networking for the rapid identification of antimicrobial metabolites from *Fusarium petroliphilum*. Front. Mol. Biosci..

[13] Baker R.A., Tatum J.H., Nemec S. (1990). Antimicrobial activity of naphthoquinones from Fusaria. Mycopathologia.

[14] Kornsakulkarn J., Dolsophon K., Boonyuen N., Boonruangprapa T., Rachtawee P., Prabpai S., Kongsaeree P., Thongpanchang C. (2011). Dihydronaphthalenones from endophytic fungus *Fusarium* sp. BCC14842. Tetrahedron.

[15] Shah A., Rather M.A., Hassan Q.P., Aga M.A., Mushtaq S., Shah A.M., Hussain A., Baba S.A., Ahmad Z. (2017). Discovery of anti-microbial and anti-tubercular molecules from *Fusarium solani*: An endophyte of *Glycyrrhiza glabra*. J. Appl. Microbiol..

[16] Chen C., Luo X., Li K., Guo C., Li J., Lin X. (2019). Antibacterial secondary metabolites from a marine sponge-derived fungus *Fusarium equiseti* SCSIO 41019. Chin. J. Antibiot..

[17] Wang Q.X., Li S.F., Zhao F., Dai H.Q., Bao L., Ding R., Gao H., Zhang L.X., Wen H.A., Liu H.W. (2011). Chemical constituents from endophytic fungus *Fusarium oxysporum*. Fitoterapia.

[18] Mohamed G.A., Ibrahim S.R.M., Alhakamy N.A., Aljohani O.S. (2022). Fusaroxazin, a novel cytotoxic and antimicrobial xanthone derivative from *Fusarium oxysporum*. Nat. Prod. Res..

[19] Renner M.K., Jensen P.R., Fenical W. (1998). Neomangicols: Structures and absolute stereochemistries of unprecedented halogenated sesterterpenes from a marine fungus of the genus *Fusarium*. J. Org. Chem..

[20] Li S., Shao M.-W., Lu Y.-H., Kong L.-C., Jiang D.-H., Zhang Y.-L. (2014). Phytotoxic and antibacterial metabolites from *Fusarium proliferatum* ZS07 Isolated from the gut of long-horned grasshoppers. J. Agric. Food Chem..

[21] Jiang C.X., Li J., Zhang J.M., Jin X.J., Yu B., Fang J.G., Wu Q.X. (2019). Isolation, identification, and activity evaluation of chemical constituents from soil fungus *Fusarium avenaceum* SF-1502 and endophytic fungus *Fusarium proliferatum* AF-04. J. Agric. Food Chem..

[22] Hussain H., Drogies K.-H., Al-Harrasi A., Hassan Z., Shah A., Rana U.A., Green I.R., Draeger S., Schulz B., Krohn K. (2015). Antimicrobial constituents from endophytic fungus *Fusarium* sp.. Asian Pac. J. Trop. Dis..

[23] Lu W., Zhu G., Yuan W., Han Z., Dai H., Basiony M., Zhang L., Liu X., Hsiang T., Zhang J. (2021). Two novel aliphatic unsaturated alcohols isolated from a pathogenic fungus *Fusarium proliferatum*. Synth. Syst. Biotechnol..

[24] Wen H., Li Y., Liu X., Ye W., Yao X., Che Y. (2015). Fusagerins A-F, new alkaloids from the fungus *Fusarium* sp.. Nat. Prod. Bioprospect..

[25] Kılıç G., Tosun G., Bozdeveci A., Erik İ., Öztürk E., Reis R., Sipahi H., Cora M., Karaoğlu Ş.A., Yaylı N. (2021). Antimicrobial, cytotoxic, antiviral wffects, and apectroscopic characterization of metabolites produced by *fusarium oxysporum* YP9B. Rec. Nat. Prod..

[26] Valla A., Giraud M., Labia R., Morand A. (1997). In vitro inhibitory activity against bacteria of a fusarium mycotoxin and new synthetic derivatives. Bull. Soc. Chim. Fr..

[27] Kyekyeku J.O., Kusari S., Adosraku R.K., Bullach A., Golz C., Strohmann C., Spiteller M. (2017). Antibacterial secondary metabolites from an endophytic fungus, *Fusarium solani* JK10. Fitoterapia.

[28] Sibero M.T., Zhou T., Fukaya K., Urabe D., Radjasa O.K.K., Sabdono A., Trianto A., Igarashi Y. (2019). Two new aromatic polyketides from a sponge-derived *Fusarium*. Beilstein. J. Org. Chem..

[29] Tsuchinari M., Shimanuki K., Hiramatsu F., Murayama T., Koseki T., Shiono Y. (2007). Fusapyridons A and B, novel pyridone alkaloids from an endophytic fungus, *Fusarium* sp. YG-45. Z. Naturforsch. B..

[30] Supratman U., Hirai N., Sato S., Watanabe K., Malik A., Annas S., Harneti D., Maharani R., Koseki T., Shiono Y. (2021). New naphthoquinone derivatives from *Fusarium napiforme* of a mangrove plant. Nat. Prod. Res..

[31] Khan N., Afroz F., Begum M.N., Roy Rony S., Sharmin S., Moni F., Mahmood Hasan C., Shaha K., Sohrab M.H. (2018). Endophytic *Fusarium solani*: A rich source of cytotoxic and antimicrobial napthaquinone and aza-anthraquinone derivatives. Toxicol. Rep..

[32] Haraguchi H., Yokoyama K., Oike S., Ito M., Nozaki H. (1997). Respiratory stimulation and generation of superoxide radicals in *Pseudomonas aeruginosa* by fungal naphthoquinones. Arch. Microbiol..

[33] Yan C., Liu W., Li J., Deng Y., Chen S., Liu H. (2018). Bioactive terpenoids from Santalum album derived endophytic fungus *Fusarium* sp. YD-2. RSC Adv..

[34] Zhang J., Liu D., Wang H., Liu T., Xin Z. (2014). Fusartricin, a sesquiterpenoid ether produced by an endophytic fungus *Fusarium tricinctum* Salicorn 19. Eur. Food Res. Technol..

[35] Dong J.W., Cai L., Li X.J., Duan R.T., Shu Y., Chen F.Y., Wang J.P., Zhou H., Ding Z.T. (2016). Production of a new tetracyclic triterpene sulfate metabolite sambacide by solid-state cultivated *Fusarium sambucinum* B10.2 using potato as substrate. Bioresour. Technol..

[36] Shu R., Wang F., Yang Y., Liu Y., Tan R. (2004). Antibacterial and xanthine oxidase inhibitory cerebrosides from *Fusarium* sp. IFB-121, and endophytic fungus in Quercus variabilis. Lipids.

[37] Sebastià N., Meca G., Soriano J.M., Mañes J. (2011). Antibacterial effects of enniatins J(1) and J(3) on pathogenic and lactic acid bacteria. Food Chem. Toxicol..

[38] Chen C., Imamura N., Nishijima M., Adachi K., Sakai M., Sano H. (1996). Halymecins, new antimicroalgal substances produced by fungi isolated from marine algae. J. Antibiot..

[39] Shiono Y., Shibuya F., Murayama T., Koseki T., Poumale H.M.P., Ngadjui B.T. (2013). A polyketide metabolite from an endophytic *Fusarium equiseti* in a medicinal plant. Z. Naturforsch. B..

[40] Ivanov M., Ćirić A., Stojković D. (2022). Emerging antifungal targets and strategies. Int. J. Mol. Sci..

[41] Van Daele R., Spriet I., Wauters J., Maertens J., Mercier T., Van Hecke S., Brüggemann R. (2019). Antifungal drugs: What brings the future?. Med. Mycol..

[42] Campoy S., Adrio J.L. (2017). Antifungals. Biochem. Pharmacol..

[43] Jackson M., Frost D.J., Karwowski J.P., Humphrey P.E., Dahod S.K., Choi W.S., Brandt K., Malmberg L.-H., Rasmussen R.R., Scherr M.H. (1995). Fusacandins A and B; Novel Antifungal Antibiotics of the Papulacandin Class from *Fusarium sambucinum* I. Identity of the Producing Organism, Fermentation and Biological Activity. J. Antibiot..

[44] Chen R.H., Tennant S., Frost D., O’Beirne M.J., Karwowski J.P., Humphrey P.E., Malmberg L.-H., Choi W., Brandt K.D., West P. (1996). Discovery of saricandin, a novel papulacandin, from a *Fusarium* species. J. Antibiot..

[45] Brady S.F., Clardy J. (2000). CR377, a new pentaketide antifungal agent isolated from an endophytic fungus. J. Nat. Prod..

[46] Saetang P., Rukachaisirikul V., Phongpaichit S., Sakayaroj J., Shi X., Chen J., Shen X. (2016). β-Resorcylic macrolide and octahydronaphthalene derivatives from a seagrass-derived fungus *Fusarium* sp. PSU-ES123. Tetrahedron.

[47] Hiramatsu F., Miyajima T., Murayama T., Takahashi K., Koseki T., Shiono Y. (2006). Isolation and structure elucidation of neofusapyrone from a marine-derived *Fusarium* species, and structural revision of fusapyrone and deoxyfusapyrone. J. Antibiot..

[48] Xu X., Zhao S., Yu Y., Chen Z., Shen H., Zhou L. (2016). Beauvericin K, a new antifungal beauvericin analogue from a marine-derived *Fusarium* sp.. Nat. Prod. Commun..

[49] Ibrahim S.R.M., Abdallah H.M., Elkhayat E.S., Al Musayeib N.M., Asfour H.Z., Zayed M.F., Mohamed G.A. (2018). Fusaripeptide A: New antifungal and anti-malarial cyclodepsipeptide from the endophytic fungus *Fusarium* sp.. J. Asian Nat. Prod. Res..

[50] Dreyfuss M., Härri E., Hofmann H.e.a., Kobel H., Pache W., Tscherter H. (1976). Cyclosporin A and C: New metabolites from *Trichoderma polysporum* (Link ex Pers.) *Rifai*. Appl. Microbiol. Biot..

[51] Baráth Z., Baráthová H., Betina V., Nemec P. (1974). Ramihyphins—Antifungal and morphogenic antibiotics from *Fusarium* sp. S-435. Folia. Microbiol..

[52] Parish C.A., Smith S.K., Calati K., Zink D., Wilson K., Roemer T., Jiang B., Xu D., Bills G., Platas G. (2008). Isolation and structure elucidation of parnafungins, antifungal natural products that inhibit mRNA polyadenylation. J. Am. Chem. Soc..

[53] Overy D., Calati K., Kahn J.N., Hsu M.J., Martin J., Collado J., Roemer T., Harris G., Parish C.A. (2009). Isolation and structure elucidation of parnafungins C and D, isoxazolidinone-containing antifungal natural products. Bioorg. Med. Chem. Lett..

[54] McBrien K.D., Gao Q., Huang S., Klohr S.E., Wang R.R., Pirnik D.M., Neddermann K.M., Bursuker I., Kadow K.F., Leet J.E. (1996). Fusaricide, a new cytotoxic *N*-hydroxypyridone from *Fusarium* sp.. J. Nat. Prod..

[55] Hilário F., Chapla V., Araujo A., Sano P., Bauab T., dos Santos L. (2016). Antimicrobial Screening of Endophytic Fungi Isolated from the Aerial Parts of *Paepalanthus chiquitensis* (Eriocaulaceae) Led to the Isolation of Secondary Metabolites Produced by *Fusarium fujikuroi*. J. Braz. Chem. Soc..

[56] Ibrahim S.M., Mohamed G., Khayat M., Al Haidari R., El-Kholy A., Zayed M. (2019). A new antifungal aminobenzamide derivative from the endophytic fungus *Fusarium* sp.. Pharmacogn. Mag..

[57] Sakai K., Unten Y., Iwatsuki M., Matsuo H., Fukasawa W., Hirose T., Chinen T., Nonaka K., Nakashima T., Sunazuka T. (2019). Fusaramin, an antimitochondrial compound produced by *Fusarium* sp., discovered using multidrug-sensitive *Saccharomyces cerevisiae*. J. Antibiot..

[58] Woscholski R., Kodaki T., McKinnon M., Waterfield M.D., Parker P.J. (1994). A comparison of demethoxyviridin and wortmannin as inhibitors of phosphatidylinositol 3-kinase. FEBS Lett..

[59] Pedersen P.B., Miller J.D. (1999). The fungal metabolite culmorin and related compounds. Nat. Toxins.

[60] Strongman D., Miller J., Calhoun L., Findlay J., Whitney N. (1987). The biochemical basis for interference competition among some lignicolous marine fungi. Bot. Mar..

[61] Kurobane I., Zaita N., Fukuda A. (1986). New metabolites of *Fusarium martii* related to dihydrofusarubin. J. Antibiot..

[62] Limón M.C., Rodríguez-Ortiz R., Avalos J. (2010). Bikaverin production and applications. Appl. Microbiol. Biotechnol..

[63] Deshmukh R., Mathew A., Purohit H.J. (2014). Characterization of antibacterial activity of bikaverin from *Fusarium* sp. HKF15. J. Biosci. Bioeng..

[64] Bushnell G.W., Li Y.-L., Poulton G.A. (1984). Pyrones. X. Lateropyrone, a new antibiotic from the fungus *Fusarium lateritium* Nees. Can. J. Chem..

[65] Clark T.N., Carroll M., Ellsworth K., Guerrette R., Robichaud G.A., Johnson J.A., Gray C.A. (2018). Antibiotic mycotoxins from an endophytic *Fusarium acuminatum* isolated from the medicinal plant *Geum macrophyllum*. Nat. Prod. Commun..

[66] Ariantari N.P., Frank M., Gao Y., Stuhldreier F., Kiffe-Delf A.-L., Hartmann R., Höfert S.-P., Janiak C., Wesselborg S., Müller W.E.G. (2021). Fusaristatins D–F and (7*S*,8*R*)-(−)-chlamydospordiol from *Fusarium* sp. BZCB-CA, an endophyte of *Bothriospermum chinense*. Tetrahedron.

[67] Ola A.R.B., Thomy D., Lai D., Brötz-Oesterhelt H., Proksch P. (2013). Inducing secondary metabolite production by the endophytic fungus *Fusarium tricinctum* through coculture with *Bacillus subtilis*. J. Nat. Prod..

[68] Okada H., Nagashima M., Suzuki H., Nakajima S., Kojiri K., Suda H. (1996). BE-29602, a new member of the papulacandin family. J. Antibiot..

[69] Kobayashi H., Sunaga R., Furihata K., Morisaki N., IWasaki S. (1995). Isolation and structures of an antifungal antibiotic, fusarielin A, and related compounds produced by a *Fusarium* sp.. J. Antibiot..

[70] Liang X.A., Ma Y.M., Zhang H.C., Liu R. (2016). A new helvolic acid derivative from an endophytic *Fusarium* sp. of Ficus carica. Nat. Prod. Res..

[71] Janevska S., Arndt B., Niehaus E.-M., Burkhardt I., Rösler S.M., Brock N.L., Humpf H.-U., Dickschat J.S., Tudzynski B. (2016). Gibepyrone biosynthesis in the rice pathogen *Fusarium fujikuroi* is facilitated by a small polyketide synthase gene cluster. J. Biol. Chem..

[72] Zhou G., Qiao L., Zhang X., Sun C., Che Q., Zhang G., Zhu T., Gu Q., Li D. (2019). Fusaricates H-K and fusolanones A-B from a mangrove endophytic fungus *Fusarium solani* HDN15-410. Phytochemistry.

[73] Evidente A., Conti L., Altomare C., Bottalico A., Sindona G., Segre A.L., Logrieco A. (1994). Fusapyrone and deoxyfusapyrone, two antifungal α-pyrones from *Fusarium semitectum*. Nat. Toxins.

[74] Altomare C., Perrone G., Zonno M.C., Evidente A., Pengue R., Fanti F., Polonelli L. (2000). Biological characterization of fusapyrone and deoxyfusapyrone, two bioactive secondary metabolites of *Fusarium semitectum*. J. Nat. Prod..

[75] Son S., Kim H., Choi G., Lim H., Jang K., Lee S., Lee S., Sung N., Kim J.C. (2008). Bikaverin and fusaric acid from *Fusarium oxysporum* show antioomycete activity against *Phytophthora infestans*. J. Appl. Microbiol..

[76] Bacon C.W., Hinton D.M., Hinton A. (2006). Growth-inhibiting effects of concentrations of fusaric acid on the growth of *Bacillus mojavensis* and other biocontrol *Bacillus* species. J. Appl. Microbiol..

[77] Poleto L., da Rosa L.O., Fontana R.C., Rodrigues E., Poletto E., Baldo G., Paesi S., Sales-Campos C., Camassola M. (2021). Production of antimicrobial metabolites against pathogenic bacteria and yeasts by *Fusarium oxysporum* in submerged culture processes. Bioproc. Biosyst. Eng..

[78] Vesonder R.F., Tjarks L.W., Rohwedder W.K., Burmeister H.R., Laugal J.A. (1979). Equisetin, an antibiotic from *Fusarium equisetin* NRRL 5537, identified as a derivative of *N*-methyl-2, 4-pyrollidone. J. Antibiot..

[79] Ratnaweera P.B., de Silva E.D., Williams D.E., Andersen R.J. (2015). Antimicrobial activities of endophytic fungi obtained from the arid zone invasive plant Opuntia dillenii and the isolation of equisetin, from endophytic *Fusarium* sp.. BMC Complement. Altern. Med..

[80] Ibrahim S.R.M., Elkhayat E.S., Mohamed G.A.A., Fat’hi S.M., Ross S.A. (2016). Fusarithioamide A, a new antimicrobial and cytotoxic benzamide derivative from the endophytic fungus *Fusarium chlamydosporium*. Biochem. Biophys. Res. Commun..

[81] Ibrahim S.R.M., Mohamed G.A., Al Haidari R.A., Zayed M.F., El-Kholy A.A., Elkhayat E.S., Ross S.A. (2018). Fusarithioamide B, a new benzamide derivative from the endophytic fungus *Fusarium chlamydosporium* with potent cytotoxic and antimicrobial activities. Bioorg. Med. Chem..

[82] Jiang Z., Barret M.-O., Boyd K.G., Adams D.R., Boyd A.S., Burgess J.G. (2002). JM47, a cyclic tetrapeptide HC-toxin analogue from a marine *Fusarium* species. Phytochemistry.

[83] Roig M., Meca G., Marin R., Ferrer E., Manes J. (2014). Antibacterial activity of the emerging *Fusarium* mycotoxins enniatins A, A(1), A(2), B, B(1), and B(4) on probiotic microorganisms. Toxicon.

[84] Meca G., Sospedra I., Valero M.A., Manes J., Font G., Ruiz M.J. (2011). Antibacterial activity of the enniatin B, produced by *Fusarium tricinctum* in liquid culture, and cytotoxic effects on Caco-2 cells. Toxicol. Mech. Method..

[85] Meca G., Soriano J.M., Gaspari A., Ritieni A., Moretti A., Manes J. (2010). Antifungal effects of the bioactive compounds enniatins A, A(1), B, B(1). Toxicon.

[86] Tsantrizos Y.S., Xu X.-J., Sauriol F., Hynes R.C. (1993). Novel quinazolinones and enniatins from *Fusarium lateritium* Nees. Can. J. Chem..

[87] Meca G., Sospedra I., Soriano J.M., Ritieni A., Moretti A., Manes J. (2010). Antibacterial effect of the bioactive compound beauvericin produced by *Fusarium proliferatum* on solid medium of wheat. Toxicon.

[88] Wu Q., Patocka J., Nepovimova E., Kuca K. (2018). A Review on the Synthesis and Bioactivity Aspects of Beauvericin, a *Fusarium* Mycotoxin. Front. Pharmacol..

[89] Hawas U.W., Al-Farawati R., Abou El-Kassem L.T., Turki A.J. (2016). Different Culture Metabolites of the Red Sea Fungus *Fusarium equiseti* Optimize the Inhibition of Hepatitis C Virus NS3/4A Protease (HCV PR). Mar. Drugs.

[90] Tompa D.R., Immanuel A., Srikanth S., Kadhirvel S. (2021). Trends and strategies to combat viral infections: A review on FDA approved antiviral drugs. Int. J. Biol. Macromol..

[91] Chang S., Yan B., Chen Y., Zhao W., Gao R., Li Y., Yu L., Xie Y., Si S., Chen M. (2022). Cytotoxic hexadepsipeptides and anti-coronaviral 4-hydroxy-2-pyridones from an endophytic *Fusarium* sp.. Front. Chem..

[92] McKee T.C., Bokesch H.R., McCormick J.L., Rashid M.A., Spielvogel D., Gustafson K.R., Alavanja M.M., Cardelline J.H., Boyd M.R. (1997). Isolation and characterization of new anti-HIV and cytotoxic leads from plants, marine, and microbial organisms. J. Nat. Prod..

[93] Guo Y.W., Liu X.J., Yuan J., Li H.J., Mahmud T., Hong M.J., Yu J.C., Lan W.J. (2020). l-Tryptophan induces a marine-derived *Fusarium* sp. to produce indole alkaloids with activity against the Zika virus. J. Nat. Prod..

[94] Nonaka K., Chiba T., Suga T., Asami Y., Iwatsuki M., Masuma R., Ōmura S., Shiomi K. (2015). Coculnol, a new penicillic acid produced by a coculture of *Fusarium solani* FKI-6853 and *Talaromyces* sp. FKA-65. J. Antibiot..

[95] Lee S.M., Kim M.S., Hayat F., Shin D. (2019). Recent Advances in the Discovery of Novel Antiprotozoal Agents. Molecules.

[96] Trisuwan K., Khamthong N., Rukachaisirikul V., Phongpaichit S., Preedanon S., Sakayaroj J. (2010). Anthraquinone, cyclopentanone, and naphthoquinone derivatives from the sea fan-derived fungi *Fusarium* spp. PSU-F14 and PSU-F135. J. Nat. Prod..

[97] Balan J., Fuska J., Kuhr I., Kuhrová V. (1970). Bikaverin, an antibiotic from *Gibberella fujikuroi*, effective against *Leishmania brasiliensis*. Folia Microbiol..

[98] Nascimento A.M.d., Conti R., Turatti I.C., Cavalcanti B.C., Costa-Lotufo L.V., Pessoa C., Moraes M.O.d., Manfrim V., Toledo J.S., Cruz A.K. (2012). Bioactive extracts and chemical constituents of two endophytic strains of *Fusarium oxysporum*. Rev. Bras. Farmacogn..

[99] Campos F.F., Sales Junior P.A., Romanha A.J., Araújo M.S., Siqueira E.P., Resende J.M., Alves T., Martins-Filho O.A., Santos V.L.d., Rosa C.A. (2015). Bioactive endophytic fungi isolated from *Caesalpinia echinata* Lam. (Brazilwood) and identification of beauvericin as a trypanocidal metabolite from *Fusarium* sp.. Mem. Inst. Oswaldo Cruz.

[100] Ibrahim S.R., Abdallah H.M., Mohamed G.A., Ross S.A. (2016). Integracides H-J: New tetracyclic triterpenoids from the endophytic fungus *Fusarium* sp.. Fitoterapia.

[101] Abdelhakim I., Bin Mahmud F., Motoyama T., Futamura Y., Takahashi S., Osada H. (2021). Dihydrolucilactaene, a potent antimalarial compound from *Fusarium* sp. RK97-94. J. Nat. Prod..

[102] Kato S., Motoyama T., Futamura Y., Uramoto M., Nogawa T., Hayashi T., Hirota H., Tanaka A., Takahashi-Ando N., Kamakura T. (2020). Biosynthetic gene cluster identification and biological activity of lucilactaene from *Fusarium* sp. RK97-94. Biosci. Biotechnol. Biochem..

[103] Abdelhakim I.A., Motoyama T., Nogawa T., Mahmud F.B., Futamura Y., Takahashi S., Osada H. (2022). Isolation of new lucilactaene derivatives from P450 monooxygenase and aldehyde dehydrogenase knockout *Fusarium* sp. RK97-94 strains and their biological activities. J. Antibiot..

[104] Singh S.B., Zink D.L., Polishook J.D., Dombrowski A.W., Darkin-Rattray S.J., Schmatz D.M., Goetz M.A. (1996). Apicidins: Novel cyclic tetrapeptides as coccidiostats and antimalarial agents from *Fusarium pallidoroseum*. Tetrahedron Lett..

[105] Singh S.B., Zink D.L., Liesch J.M., Dombrowski A.W., Darkin-Rattray S.J., Schmatz D.M., Goetz M.A. (2001). Structure, histone deacetylase, and antiprotozoal activities of apicidins B and C, congeners of apicidin with proline and valine substitutions. Org. Lett..

[106] Von Bargen K.W., Niehaus E.-M., Bergander K., Brun R., Tudzynski B., Humpf H.-U. (2013). Structure elucidation and antimalarial activity of apicidin F: An apicidin-like compound produced by *Fusarium fujikuroi*. J. Nat. Prod..

[107] Liu Y., Xu M., Tang Y., Shao Y., Wang H., Zhang H. (2022). Genome features and antiSMASH analysis of an endophytic strain *Fusarium* sp. R1. Metabolites.

[108] Zhang H., Zhang C., Li Q., Ma J., Ju J. (2022). Metabolic blockade-based genome mining reveals lipochain-linked dihydro-*β*-alanine synthetases involved in autucedine biosynthesis. Org. Lett..

[109] Kang H.S., Charlop-Powers Z., Brady S.F. (2016). Multiplexed CRISPR/Cas9- and TAR-mediated promoter engineering of natural product biosynthetic gene clusters in yeast. ACS Synth. Biol..

[110] Keller N.P. (2019). Fungal secondary metabolism: Regulation, function and drug discovery. Nat. Rev. Microbiol..

