# Supplementary material for: Fusarium-Derived Secondary Metabolites with Antimicrobial Effects
Source: Molecules. 2023 Apr 13;28(8):3424. doi: 10.3390/molecules28083424 (PMC10142451; doi:10.3390/molecules28083424)
Supplement: Supplementary file 1 [file molecules-28-03424-s001.zip › molecules-2332316-supplementary.pdf]

*Supplementary materials for*

# *Fusarium*-derived secondary metabolites with antimicrobial effects

**Meijie Xu, Ziwei Huang, Wangjie Zhu, Yuanyuan Liu, Xuelian Bai * and Huawei Zhang ***

## Contents

**Table S1.** Detail information for *Fusarium*-derived anti-Gram-positive bacterial SMs----------2

**Table S2**. Detail information for *Fusarium*-derived anti-Gram-negative bacterial SMs----------5

**Table S3**. Detail information for *Fusarium*-derived both anti-Gram-positive and anti-Gram-negative SMs----------6

**Table S4**. Detail information for *Fusarium*-derived antifungal SMs----------8

**Table S5**. Detail information for *Fusarium*-derived both antibacterial and antifungal SMs----------10

**Table S6**. Detail information for *Fusarium*-derived antiviral SMs----------14

**Table S7**. Detail information for *Fusarium*-derived antiparasitic SMs----------15

References----------17

Table S1. Detail information for *Fusarium*-derived anti-Gram-positive bacterial SMs.

| No. | Name | Strain | Source | Bioactivity | Ref. |
|---|---|---|---|---|---|
| **1** | fusariumins C | *F. oxysporum* ZZP-R1 | the coastal plant *R. madaio* Makino, China | potent activity against *S. aureus* with an MIC value of 6.25 μM | [1] |
| **2** | fusariumins D | | | moderate inhibitory effect on *S. aureus* with an MIC value of 25.0 μM | |
| **3** | A-108835 | *F. compactu* AB 21941-103 | an ant hill soil sample, Nigeria | only scattered weak Gram-positive activity against some *S. aureus* and *Streptococcus* strains in the range of 6~50 μg/mL | [2] |
| **4** | A-108836 | | | | |
| **5** | enniatin Q | *F. tricinctum* Corda | the fruits of *Hordeum sativum* Jess | mild activity against *S. aureus* | [3] |
| **6** | enniatin E | *Fusarium* sp. FO-1305 | Soil, Japan | weak activity against Gram-positive bacteria such as *S. aureus*, *B. subtilis*, *B. cereus* and *M. luteus* and very weak activity against *C. albicans* and *P. oryzae*. | [4] |
| **7** | enniatin F | | | | |
| **8** | enniatin H | *Fusarium* sp. TP-G1 | the root of *Dendrobium officinale* Kimura et Migo | weak antibacterial activities against *S. aureus* and MRSA with MIC value of 32 μg/mL | [5] |
| **9** | enniatin I | | | weak antibacterial activities against *S. aureus* and MRSA with MICs of 8 μg/mL and 16 μg/mL, respectively | |
| **10** | enniatin MK1688 | | | | |
| **11** | beauvericin A | | | good antibacterial activities against *S. aureus* and MRSA with MIC value of 2.0 μg/mL | |
| **12** | trichosetin | | | | |
| **13** | *epi*-trichosetin | *F. oxysporum* FKI-4553 | - | antimicrobial activity, particularly against Gram-positive bacteria, including methicillin-sensitive and -resistant *S. aureus* | [6] |
| **14** | lateritin | *Fusarium* sp. 2TnP1-2 | The petioles of *Trewia nudiflora* L. (Euphorbiaceae), China | possess antibacterial activity against *Staphylococcus aureus* | [7] |
| **15** | oxysporizoline | *F. oxysporum* | the marine mudflat, Korea | weak antibacterial activity against MRSA and MDRSA, with MICs of 6.25 μg/mL | [8] |
| **16** | bromomethylchlamydosporol A | *F. tricinctum* | the edible marine brown alga *Sargassum ringgoldium*, Korea | an MIC of 15.6 μg/mL against *S. aureus*, MRSA, and MDRSA (mild antibacterial activity) | [9] |
| **17** | bromomethylchlamydosporol B | | | | |
| **18** | chlamydosporol | *F. oxysporum* | the marine mudflat, Korea | weak antibacterial activity against MRSA and MDRSA, with MICs of 31.5 μg/mL | [8] |
| | | *F. tricinctum* | the edible marine brown alga *Sargassum ringgoldium*, Korea | an MIC of 31.5 μg/mL against *S. aureus* and 62.5 μg/mL against MRSA | [9] |
| **19** | 4-methoxy-6-((*E*)-4,6-dimethyloct-2-en-2-yl) -2*H*-pyran-2-one | *F. Petroliphilum* FEP 16 | *Posidonia oceanica* shoots, France | moderate activity against MRSA with an MIC of 32 μg/mL | [10] |
| **20** | trans-Dihydrofusarubin | *F. solani* B3H, B3K, and BP15-8 (NRRL 15980) | fibrous roots of citrus trees | significant activity against *S. aureus* at MIC values <4 μg/mL | [11] |
| **21** | 3-*O*, 9-*O*-Metylfusarubin | | | | |
| **22** | 5-*O*-Methyljavanicin | | | | |
| **23** | anhydrofusarubin | *F. solani* B3H, B3K, and BP15-8 (NRRL 15980) | fibrous roots of citrus trees | significant activity against *S. aureus* at MIC values <4 μg/mL | [11] |
| | | *Fusarium* sp.PSU-F135 | Marine brown alga *Colpomenia sinuosa* | antimycobacterial (against *M. tuberculosis* H37Ra, MIC 87 μM) activities | [12] |

| | | | | | |
|---|---|---|---|---|---|
| **24** | 5,8-Dihydroxy-2-methoxy-6-hydroxymethyl-7-(2-hydroxypropyl)-1,4-naphthalenedione | *F. solani* B3H, B3K, and BP15-8 (NRRL 15980) | fibrous roots of citrus trees | significant activity against *S. aureus* at MIC values <4 μg/mL | [11] |
| **25** | 8-*O*-Methyljavanicin | *F. oxysporum* HC-7-1 (ATCC 64432) | *Hyrtios proteus* sponge | significant activity against *S. aureus* at MIC values <4 μg/mL | [11] |
| **26** | 2,3-Dihydro-5,8-dihydroxy-6-methoxy-2-hydroxymethyl-3-(2-hydroxypropyl)- 1,4-naphthalenedione | *F. solani* B3H, B3K, and BP15-8 (NRRL 15980) | fibrous roots of citrus trees | significant activity against *S. aureus* and *S. pyogenes*, at MIC values <4 μg/mL | [11] |
| **27** | 2,3-Dihydro-5-hydroxy-4-hydroxymethyl-8-methoxy-3-methylnaphtho(1,2-b) furan-6,9-dione | | | significant activity against *Staphylococcus aureus* and *S. pyogenes*, at MIC values <4 μg/mL; | [11] |
| **28** | 9*α*-hydroxyhalorosellinia A | *Fusarium* sp. PSU-F14 | a gorgonian sea fan | antimycobacterial (against *M. tuberculosis* H37Ra, MIC 38.57 μM) activity | [12] |
| **29** | nigrosporin B | | | antimycobacterial (against *M. tuberculosis* H37Ra, MIC 41 μM) activity | [12] |
| **30** | 5-hydroxy-3-methoxydihydrofusarubin A | *Fusarium* sp. BCC14842 | Bamboo leaf, Thailand | anti-TB (*M. tuberculosis*) with MIC of 50 μg/mL | [13] |
| **31** | dihydronaphthalenone diastereomer | | | anti-TB (*M. tuberculosis*) with MIC of 25 μg/mL | [13] |
| **32** | 3-*O*-methylfusarubin | *Fusarium* sp. BCC14842 | - | anti-TB (*M. tuberculosis*) with MIC of 50 μg/mL | [13] |
| | | *F. solani* A2 | | | |
| **33** | 3,6,9-trihydroxy-7-methoxy-4,4-dimethyl-3,4-dihydro-1*H*-benzo[g]isochromene-5,10-dione | *F. solani* A2 | medicinal plant *Glycyrrhiza glabra*, India | moderate activity against *M. tuberculosis strain* H37Rv with MIC value of 64 μg/mL; moderate anti-bacterial activity against *S. aureus* with MIC value of 32 μg/mL; against *B. cereus* and *S. pyogenes* with MIC of <1 μg/mL as compared to ciprofloxacin whose MIC against these strains is 0.15 and 10 μg/mL, respectively | [14] |
| **34** | linoleic acid | *F. equiseti* SCSIO 41019 | Sponge (*Callyspongia* sp.), China | weak antibacterial activities against SA and MRSA with MIC values of 62.5, 125 μg/mL, respectively | [15] |
| **35** | *epi*-equisetin | *F. equiseti* SCSIO 41019 | Sponge (*Callyspongia* sp.), China | strongest antibacterial activities against *S. aureus* and MRSA with an MIC value of 31.2 μg/mL | [15] |
| | | *F. equiseti* AGR12 | the stem of *Rhizophora stylosa* Griff | remarkable antimicrobial activities against *B. subtilis and S. aureus*, with MIC value of 32 μg/mL | [16] |
| **36** | (-)-4, 6′-anhydrooxysporidinone | *F. oxysporum* | the bark of *C. kanehirae*, Taiwan Province | showed weak anti-MRSA activity (MIC=100 μg/mL) and moderate anti-BS (*B. subtilis*) activity (MIC=25 μg/mL). | [17] |
| **37** | fusaroxazin | *F. oxysporum* | *V. faba*, Egypt | significant antibacterial activity towards *S. aureus* (IZD 14.8 mm and MIC 5.3 μg/mL) and *B. cereus* (IZD 18.9 mm and MIC 3.7 μg/mL), in comparison to ciprofloxacin (IZDs 16.9 and 20.5 mm; MICs 3.9 and 2.3 μg/mL, respectively) | [18] |
| **38** | neomangicol B | *Fusarium* sp. CNC-477 | the surface of driftwood collected in the Bahamas Islands | antibacterial activity similar to that of the known antibiotic, gentamycin, against the Gram-positive bacterium *B. subtilus* | [19] |
| **39** | SMA93 | *F. proliferatum* ZS07 | *Longhorned grasshoppers,* | antibacterial effect against *B. subtilis* with MIC value of 6.25 μg/mL | [20] |

| | | | | | |
|---|---|---|---|---|---|
| **40** | 6-*O*-methyl SMA93 | | China | antibacterial effect against *B. subtilis* with MIC value of 12.5 µg/mL | |
| **41** | rhodolamprometrin | | | antibacterial effect against *B. subtilis* with ZOI of 24.8mm and MIC value of 3.13 µg/mL | |
| **42** | cyclonerodiol | *F. avenaceum* SF-1502 | Soil | stronger antibacterial activity against *B. megaterium* than the positive controls, the clinical drugs, ampicillin, erythromycin, and streptomycin | [21] |
| **43** | epicyclonerodiol oxide | | | against *B. megaterium* with ampicillin and erythromycin, and stronger than streptomycin | |
| **44** | 4,5-dihydroascochlorin | *Fusarium* sp. | - | activity against *B. megaterium* | [22] |
| **45** | fusariumnol A | *F. proliferatum* 13294 | wheat tissue | weak antibacterial activity against *S. epidermidis* (MIC = 100 µM). | [23] |
| **46** | fusariumnol B | | | | |
| **47** | fungerin | *Fusarium* sp. | Soil, Qinghai-Tibetan plateau | antibacterial activity against *S. aureus* and *S. pneumoniae* (with $IC_{50}$ values of 33.8 and 34.5 µM, respectively) | [24] |
| **48** | 3-hydroxy-1,2,6,10-tetramethylundecyl hexzadecanoate | *F. oxysporum* YP9B | the tomato plant root in Pazar-Rize, Turkey | against Gram-positive bacteria (*S. aureus*, *E. faecalis*, *S. mutans*, *B. cereus*, and *M. smegmatis*) | [25] |
| **49** | 3-hydroxy-1,2,6,10-tetramethylundecyl (9*E*)-octadecaenoate | | | | |
| **50** | 3-hydroxy-1,2,6,10-tetramethylundecyl-octadecanoate | | | | |

Table S2. Detail information for *Fusarium*-derived anti-Gram-negative bacterial SMs.

| No. | Name | Strain | Source | Bioactivity | Ref. |
|---|---|---|---|---|---|
| **51** | butenolide | *Fusarium* sp. | - | selective inhibitory activity against *E. coli.* | [26] |
| **52** | (3a*S*,6*R*,6a*R*)-3a,6-dihydroxy-6-((2*E*,4*E*,6*E*)-7-(4-(1-hydroxyethyl)-2-methyl-2,5-dihydrofuran-2-yl)-2-methylhepta-2,4,6-trienoyl) hexahydro -5*H*-furo[3,2-b] pyrrol-5-one | *F. solani* JK10 | the roots of *Chlorophora regia* (Moraceae), Asakraka forest in the Eastern Region of Ghana | antibacterial efficacies against the soil bacterium *Acinetobacter* sp., comparable to the reference standard streptomycin; antibacterial activity against the environmental strain of *E. coli*, 5~10 μg/mL | [27] |
| **53** | (1R,4S,5R)-4-hydroxy-4-(2-hydroxyethyl)-1-((2E,4E,6E)-7-(4-(1-hydroxyethyl)-2-methyl-2,5-dihydrofuran-2-yl)-2-methylhepta-2,4,6-trienoyl)-6-oxa-3-azabicyclo[3.1.0]hexan-2-one | | | | |
| **54** | Methyl (2*E*,3*E*,5*E*,7*E*,9*E*)-11-((3a*S*,6*S*,6a*R*)-3a,6-dihydroxy-5-oxohexahydro-2*H*-furo[3,2-b] pyrrol-6-yl)-2-ethylidene-11-hydroxy-4,10-dimethylundeca-3,5,7,9-tetraenoate | | | | |
| **55** | (1*R*,4*S*,5*R*)-4-hydroxy-4-(2-hydroxyethyl)-1-((2*E*,4*E*,6*E*,8*E*,10*E*)-10-(hydroxymethyl)-2,8-dimethyldodeca-2,4,6,8,10-pentaenoyl)-6-oxa-3-azabicyclo[3.1.0] hexan-2-one | | | antibacterial activity against the environmental strain of *E. coli*, 5~10 μg/mL | |
| **56** | (1*R*,4*S*,5*R*)-4-hydroxy-4-(2-hydroxyethyl)-1-((2*E*,4*Z*,6*E*,8*E*,10*E*)-10-(hydroxymethyl)-2,8-dimethyldodeca-2,4,6,8,10-pentaenoyl)-6-oxa-3-azabicyclo[3.1.0] hexan-2-one | | | | |
| **57** | (3a*S*,6*R*,6a*R*)-3a,6-dihydroxy-6-((2*E*,4*E*,6*E*)-7-(4-(1-hydroxyethyl)-2,3-dimethyl-5-oxo-2,5-dihydrofuran-2-yl)-2-methylhepta-2,4,6-trienoyl)-6a-methylhexahydro-5*H*-furo[3,2-b] pyrrol-5-one | | | | |
| **58** | methyl (2*E*,3*E*,5*E*,7*E*,9*E*)-2-ethylidene-11-((3*S*,5*S*)-5-(2-hydroxyethyl)- 2-oxopyrrolidin-3-yl)-4,10-dimethyl-11-oxoundeca-3,5,7,9-tetraenoate | | | | |
| **59** | NG–391 | | | | |
| **60** | NG–393 | | | | |
| **61** | karimunones B | *Fusarium* sp. KJMT.FP.4.3 | a sponge *Xestospongia sp.*, Indonesia | weak activity against multidrug resistant *Salmonella enterica* ser with an MIC value of 125 μg/mL | [28] |
| **62** | 7-*O*-methylrhodolamprometrin | | | | |
| **63** | tricinonoic acid | | | | |
| **64** | fusapyridons A | *Fusarium* sp. YG-45 | *Maackia chinensis*, China | antimicrobial activity against *P. aeruginosa* (MIC 6.25 μg/mL) and *S. aureus* (MIC 50 μg/mL) | [29] |

Table S3. Detail information for *Fusarium*-derived both anti-Gram-positive and anti-Gram-negative bacterial SMs

| No. | Name | Strain | Source | Bioactivity | Ref. |
|---|---|---|---|---|---|
| **65** | anhydrojavanicin | *F. solani* B3H, B3K, and BP15-8 (NRRL 15980) | fibrous roots of citrus trees | significant activity against *S. aureus* at MIC values <4 μg/mL | [11] |
| **66** | methyl ether fusarubin | *F. proliferatum* AF-04 | - | selective antibacterial activities against *B. megaterium*, *B. subtilis*, *C. perfringens*, *E. coli*, MRSA | [21] |
| **67** | 5-*O*-methylsolaniol | | | | |
| **68** | 5-*O*-Methyljavanicin | *F. Solani* B3H, B3K, and BP15-8 (NRRL 15980) | fibrous roots of citrus trees | significant activity against *S. aureus* at MIC values <4 μg/mL | [11] |
| **69** | 6-hydroxy-astropaquinone B | *F. napiforme* | the mangrove plant, *Rhizophora mucronata* | moderate antibacterial activity against *Staphylococcus aureus* and *Pseudomonas aeruginosa* | [30] |
| **70** | astropaquinone D | | | | |
| **71** | bostrycoidin (bostricoidin) | *F. solani* | - | moderate antibiotic activity against Gram-positive bacteria (*S. aureus*, *S. pyogenes*) | [11] |
| | | *F. solani* | fresh healthy roots of *Cassia alata* Linn. | exhibited prominent inhibition against the above tested pathogenic bacteria | [31] |
| | | *Fusarium* sp. | - | antimicrobial activity against *Pseudomonas aeruginosa* | [32] |
| **72** | fusariumin A | *Fusarium* sp. YD-2 | twigs of the *Santalum album*, China | significant activities against *S. aureus* and *P. aeruginosa* with an MIC value of 6.3 μg/mL | [33] |
| **73** | asperterpenoid A | | | moderate activities against *S. enteritidis* and *M. luteus* with MIC values of 6.3 and 25.2 μg/mL, respectively | |
| **74** | fusarielin B | *F. tricinctum* Salicorn 19 | The aerial parts of *S. bigelovii*, China. | a broader spectrum antimicrobial activity against *M. smegmati*, *B. subtilis*, *M. phlei* and *E. coli* with MIC values 19, 19, 10 and 10 μM, respectively | [34] |
| **75** | sambacide | *F. sambucinum* B10.2 | - | significant antibacterial activities against *S. aureus* and *E. coli* | [35] |
| **76** | (2*S*,2'*R*,3*R*,3'*E*,4*E*,8*E*)-1-O-b-D-glucopyranosyl-2-N-(2'hydroxy-3'octadecenoyl)-3-hydroxy-9-methyl-4,8- sphingadienine | *Fusarium* sp. IFB-121 | *Quercus variabilis* | strong antibacterial activities against *B. subtilis*, *E. coli*, and *P. fluorescens*, with MICs of 3.9, 3.9, and 1.9 μg/mL, respectively | [36] |
| **77** | (2*S*,2'*R*,3*R*,3'*E*,4*E*,8*E*,10*E*)-1-O-b-D-glucopyranosyl-2-N-(2'hydroxy-3'octadecenoyl)-3-hydroxy-9-methyl-4,8,10-sphingatrienine | | | strong antibacterial activities against *B. subtilis*, *E. coli*, and *P. fluorescens*, with MICs of 7.8, 3.9, and 7.8 μg/mL, respectively | |
| **78** | enniatin $J_1$ | *F. tricinctum* | - | antimicrobial activity against *C. perfringens*, *E. faecium*, *E. coli*, *S. dysenteriae*, *S.aureus*, *Y. enterocolitica* and lactic acid bacteria except *B.* | [37] |
| **79** | enniatin $J_3$ | | | | |

| | | | | | |
|---|---|---|---|---|---|
| | | | | *adolescentis* | |
| **80** | halymecin A | *Fusarium* sp. FE-71-1 | Marine Algae, Japan | activity against *E. faecium*, *K. pneumoniae* and *P. vulgaris* MIC 10 (µg/mL) | [38] |
| **81** | fusaequisin A | *F. equiseti* SF-3-17 | The medicinal plant, *Ageratum conyzoides* L., Cameroon | moderate antimicrobial activity against *S. aureus* NBRC 13276 and *P. aeruginosa* ATCC 15442 | [39] |

Table S4. Detail information for *Fusarium*-derived antifungal SMs

| No. | Name | Strain | Source | Bioactivity | Ref. |
|---|---|---|---|---|---|
| **82** | fusacandin A | *F. sambucinum* AB 1900A-1314 | a polypore fruitbody, U.S.A. | antifungal activity against *C. albicans* | [40] |
| **83** | fusacandin B | | | | |
| **84** | saricandin | *Fusarium sp*. AB 2202W-161 | a soil sample, Nepal | good activity against *C. albicans* | [41] |
| **85** | CR377 | *Fusarium* sp. CR377 | the interior of a *Selaginella pallescens* stem, Costa Rica | significant activity against *C. albicans* | [42] |
| **86** | zearalenone | *Fusarium sp*. PSU-ES73 | leaves of *T. hemprichii* seagrass, Thailand | weak activity against *C. neoformans* with an MIC value of 50.26 μM | [43] |
| **87** | 5'*β*-hydroxyzearalenone | *Fusarium* sp. PSU-ES123 | a seagrass, *Enhalus acoroides,* Thailand | weak antifungal activity against *C. neoformans* with an MIC value of 128 μg/mL | [44] |
| **88** | neofusapyrone | *Fusarium* sp. FH-146 | driftwood, Japan | moderate activity against *A. clavatus* F318a | [45] |
| **89** | beauvericin K | *Fusarium* sp. | seawater collected from the Bohai Sea, China | significant activity against the yeast *C. albicans* with an IC50 value of 6.25 μg/mL | [46] |
| **90** | beauvericin D | | | | |
| **91** | cyclosporins A, B and C | *Fusarium* sp. S-435 | - | activity against filamentous pathogenic and saprophytic fungi | [47] |
| **92** | | | | | |
| **93** | | *F. solani* | soil, Japan | *V. ceratosperma* and *H. oryzae* | [48] |
| **94** | fusaripeptide A | *Fusarium* sp. | the roots of *Mentha longifolia* L. (Labiatae), Saudi Arabia | potent antifungal activity toward *C. albicans*, *C. glabrata*, *C. krusei*, and *A. fumigates* with $IC_{50}$ values of 0.11, 0.24, 0.19, and 0.14 μM, respectively | [49] |
| **95**<br>**96** | parnafungin A<br>parnafungin B | *F. larvarum* F-155,597 | - | parnafungin A had in vivo efficacy in a murine model of disseminated candidiasis. | [50] |
| | | *F. larvarum* MF7022 and MF7023 | an unidentified lichen thallus, Spain | broad spectrum activity against the ascomycetous yeasts, *C. albicans* (MIC 0.014 μg/mL), *C. krusei* (0.014 μg/mL), *C. glabrata* (1.1 μg/mL), *C. tropicalis* (3.3 μg/mL), *C. lusitaniae* (1.1 μg/mL), *C. parapsilosis* (1.1 μg/mL) and *S. cereVisiae* (3.3 μg/mL). Under identical assay conditions, the MIC of caspofungin (Cancidas) against *C. albicans* was 0.01 μg/mL. The parnafungins also were active in an agar assay against *A. fumigatus*. | [51] |
| **97** | parnafungin C | *F. larvarum* F-155,597 | - | less potent against all of the *Candida* species tested, but in some cases, such as against *C. tropicalis* and *C. lusitaniae*, these analogs had comparable antifungal activity | [50] |
| **98** | parnafungin D | | | | |
| **99** | fusaricide | *Fusarium* sp. | Flowers of sourwood *(Oxydendron arboreum),* Georgia State Botanical Garden in Athens | antifungal activity against *Candida albicans* and *Penicillium chrysogenum* (MICs 16 and 8 μg/mL, respectively). | [52] |
| **100** | indole acetic acid | *F. fujikuroi* | the aerial parts of *Paepalanthus chiquitensis* (Eriocaulaceae), | moderate antimicrobial activity for all the bacterial strains (*S. aureus, E. coli, S. setubal*) evaluated and activity against the fluconazole-resistant *C. albicans.* | [53] |

| | | | | | |
|---|---|---|---|---|---|
| | | | Brazil | | |
| **101** | fusaribenzamide A | *Fusarium* sp. | the internal tissue of *Mentha longifolia* roots | possessed a significant antifungal activity towards *C. albicans* with MIC 11.9 µg/disc compared to nystatin (MIC 4.9 µg/disc). However, it showed moderate activity toward *S. aureus* and *E. coli* with MIC values 62.8 and 56.4 µg/disc, respectively in comparison to ciprofloxacin (MICs 12.5 and 10.4 µg/disc, respectively). | [54] |
| **102** | sambutoxin | *Fusarium* sp. FKI-7550 | Soil, Japan | significant activities against multidrug-sensitive *S. cerevisiae* 12geneΔ0HSR-iERG6 with an MIC value of 0.064 µg/mL | [55] |
| **103** | *N*-demethylsambutoxin | | | significant activities against multidrug-sensitive *S. cerevisiae* 12geneΔ0HSR-iERG6 with an MIC value of 0.32 µg/mL | |
| **104** | 6-deoxyoxysporidinone | | | significant activities against multidrug-sensitive *S. cerevisiae* 12geneΔ0HSR-iERG6 with an MIC value of 2.0 µg/mL | |
| **105** | oxysporidinone | *F. oxysporum* N17B | a grassy area in Lakselv, Norway | selective potent activity against *C. albicans* | [56] |
| **106** | 6-*epi*-oxysporidinone | | | selective fungistatic activity against *A. fumigatus* | |
| **107** | wortmannin | | | | |
| **108** | culmorin | *Fusarium* sp. | - | both marine (*S. marina*, *M. pelagica*) and medically relevant fungi (*A. fumigatus*, *A. niger*, *C. albicans*, *Rhizopus species*, *T. mentagrophytes*) as being in the 1 µM range | [57,58] |

Table S5. Detail information for *Fusarium*-derived both antibacterial and antifungal SMs.

| No. | Name | Strain | Source | Bioactivity | Ref. |
|---|---|---|---|---|---|
| **109** | javanicin | *Fusarium* sp. BCC14842 | Bamboo leaf, Thailand | antifungal (*C. albicans*) activity with $IC_{50}$ of 6.16 µg/mL; anti-TB (*M. tuberculosis*) with MIC of 25 µg/mL | [13] |
| | | *F. solani* A2 | plant '*Glycyrrhiza glabra*', India | against *B. cereus* and *S. pyogenes* with MIC of <1 µg/mL as compared to ciprofloxacin whose MIC against these strains is 0.15 and 10 µg/mL, respectively | [14] |
| | | *F. javanicum* | - | - | [59] |
| | | *F. solani* B3H, B3K, and BP15-8 (NRRL 15980) | fibrous roots of citrus trees | significant activity against *S. aureus* at MIC values <4 µg/mL | [11] |
| **110**<br>**111** | fusarubin<br>dihydrofusarubin | *F. solani* B3H, B3K, and BP15-8 (NRRL 15980); *F. martii* | fibrous roots of citrus trees; - | significant activity against *S. aureus* at MIC values <4 µg/mL; moderate antibiotic activity against Gram-positive bacteria and fungi. (*C. albicans, S. cerevisiae, S. pneumoniae, S. pyogenes, Bacillus subtilis, S. aureus*) | [11]; [60] |
| **112** | 5,10-dihydroxy-3,7-dimethoxy-3-methyl-3,4-dihydro-1*H*-benzo[*g*]isochromene-6,9-dione | *F. martii* | - | moderate antibiotic activity against Gram-positive bacteria and fungi. (*C. albicans, S. cerevisiae, S. pneumoniae, S. pyogenes, Bacillus subtilis, S. aureus*) | [60] |
| **113** | 3-ethoxy-5,10-dihydroxy-7-methoxy-3-methyl-3,4-dihydro-1*H*-benzo[*g*]isochromene-6,9-dione | (same as above) | | | |
| **114** | (3*S*,4a*R*,10a*S*)-6,9-dihydroxy-3,7-dimethoxy-3-methyl-3,4,4a,10a-tetrahydro-1*H*-benzo[*g*]isochromene-5,10-dione | (same as above) | | | |
| **115** | (3*S*,4a*R*,10a*S*)-3-ethoxy-6,9-dihydroxy-7-methoxy-3-methyl-3,4,4a,10a-tetrahydro-1*H*-benzo[*g*]isochromene-5,10-dione | (same as above) | | | |
| **116** | bikaverin | *Fusarium* sp. HKF15 | Soil, India | bikaverin inhibits *E. coli* growth significantly in the growth curve study in a microtiter plate reader | [61] |
| | | *Fusarium* sp. | - | partially inhibited the growth on agar of *Penicillium notatum*, *Alternaria humicola*, and *Aspergillus flavus* | [46,62] |
| **117** | lateropyrone | *Fusarium* sp. BZCB-CA | *Bothriospermum chinense*, China | significant inhibitory activity against MRSA and vancomycin-resistant of *E. faecalis* and *E. faecium*, with MIC values of 3.1, 12.5 and 25 µM, respectively | [63] |
| | | *F. tricinctum* | healthy rhizomes of *Aristolochia paucinervis*, Morocco | good antibacterial activity against *B. subtilis, S. aureus, S. pneumoniae, and E. faecalis,* with MIC values ranging from 2 to 8 µg/mL | [64] |
| | | *F. lateritium* Nees | *Tsuga heterophylla* (Raf.) Sarg. Trees, Canada | significant inhibitory activity towards the growth of the gram-positive bacterium *S. aureus* and the yeast *C. albicans*, but did not affect the | [65] |

| | | | | | |
|---|---|---|---|---|---|
| | | | | gram-negative bacterium *E. coli* | |
| | | *F. acuminatum* TC2-084 | The medicinal plant *Geum macrophyllum*, Canada | good activity against *M. tuberculosis* H37Ra ATCC 25177, *S. aureus* ATCC 29213, MRSA, *E. faecium* ATCC 35667, VRE, *C. albicans* ATCC 14053 | [66] |
| **118** | BE-29602 | *Fusarium sp*. AB 2202W-161 | a soil sample, Nepal | good activity against *C. albicans* | [41] |
| | | *Fusarium* sp. F29602 | a soil sample, Japan | good activity against *C. albicans, S. cerevisiae, S. pombe, P. chrysogenum*, *B. subtilis*, *B. cereus*, *M. luteus* | [67] |
| **119** | fusarielin A | *Fusarium* sp. K432 | soil | moderate antifungal activities against *P. fumigatus*, *A. kikuchiana*, *C. lindemuthianum, F. nivale*, *E. oryzae*, *P. oryzae* | [68] |
| | | *F. tricinctum* | the edible marine brown alga *Sargassum ringgoldium*, Korea | activities against *S. aureus,* MRSA and MDRSA | [9] |
| **120** | helvolic acid methyl ester | *Fusarium* sp. JX119038 | *Ficus carica*, China | potent antifungal and antibacterial activities (*B. subtilis*, *S. aureus*, *E. coli, B. cinerea*, *F. Graminearum* and *P. capsica*) | [69] |
| **121** | helvolic acid | | | | |
| **122** | hydrohelvolic acid | | | | |
| **123** | fusartricin | *F. tricinctum* Salicorn 19 | The aerial parts of *S. bigelovii*, China. | significant antimicrobial activities against *E.aerogenes*, *M. tetragenu* and *C. albicans* with the MIC values 19, 19 and 19 µM, respectively | [34] |
| **124** | gibepyrone A | *F. fujikuroi* | - | antimicrobial activity against Gram-positive bacteria (*Bacillus subtilis*, *Staphylococcus aureus*) and yeasts (*Saccharomyces cerevisiae, Candida albicans*) | [70] |
| **125** | gibepyrone B | | | | |
| **126** | fusolanone B | *F. solani* HDN15-410 | the root of *Rhizophora apiculata Blume*, China | activity with MIC value 6.25 µg/mL on *V. parahaemolyticus*. antimicrobial activity against *M. albican*, *P. aeruginosa*, *B. subtilis* and *V. parahaemolyticus* | [71] |
| **127** | fusapyrone | *Fusarium* sp. FH-146 | driftwood, Japan | weak activity against *A. clavatus* F318a and *P. aeruginosa* ATCC 15442 with MICs of 25 µg/mL and 50 µg/mL, respectively | [45] |
| | | *F. semitectum* | infected maize stalk tissues collected in southern Italy | Fusapyrone inhibits *G. Candidum* | [72] |
| | | *F. semitectum* ITEM-393 | - | antifungal activity against filamentous fungi and difficult-to-treat human pathogenic fungi such as *Aspergillus* spp.; *C. kefyr*, an emergent opportunistic pathogen, showed a remarkable sensitivity only to fusapyrone | [73] |
| **128** | deoxyfusapyrone | *Fusarium* sp. FH-146 | driftwood, Japan | moderate activity against *A. clavatus* F318a, *P. aeruginosa* ATCC 15442 | [45] |
| | | *F. semitectum* | infected maize stalk tissues collected in southern Italy | deoxyfusapyrone inhibits *G. Candidum* | [72] |
| | | *F. semitectum* ITEM-393 | - | antifungal activity against filamentous fungi and difficult-to-treat human pathogenic fungi such as *Aspergillus* spp. | [73] |

| | | | | | |
|---|---|---|---|---|---|
| **129** | fusaric acid | *Fusarium* sp. TP-G1 | the Root of *Dendrobium officinale* Kimura et Migo | antibacterial activity against *A. baumannii*, with an MIC of 64μg /mL | [5] |
| | | *F. oxysporum* EF119 | a healthy root of red pepper (*Capsicum annuum* L.), Korean | fusaric acid effectively suppressed the mycelial growth of two oomycetes, such as *P. capsici* and *P. infestans* with $IC_{50}$ values less than 1 μg/mL. Fusaric acid suppressed completely the growth of all bacteria tested at concentrations less than 100 μg/mL. The $IC_{50}$ values ranged from 0.2 to 12 μg/mL. | [74] |
| | | *F. fujikuroi* | the Aerial parts of *Paepalanthus chiquitensis* (Eriocaulaceae), Brazil | moderate antimicrobial activity for all the bacterial strains evaluated. | [53] |
| | | *F. verticillioides* RRC 408 and MRC 826 | - | inhibitory to the growth of most *Bacillus* species | [75] |
| | | *F. oxysporum* PR-33 | Torres-Rio Grande do Sul, Brazil | inhibit the growth of most *Bacillus* species | [76] |
| **130** | equisetin | *F. equiseti* NRRL 5537 | - | against several strains of Gram-positive bacteria *B. subtilis, M. phlei* and *S. aureus* and the Gram-negative bacteria *N. perflava,* at concentrations of 0.5-4.0 μg/mL of growth substrate | [77] |
| | | Coculture of *S. erythraea* with *F. pallidoroseum* | - | minimum inhibitory concentrations were <1.25 μg against *S. aureus* and 2.5 μg against *S. erythraea*. | [78] |
| | | *Fusarium* sp. | *Opuntia dillenii* | antibacterial activities against the Gram-positive bacteria *B. subtilis, S. aureus* and MRSA with MICs of 8–16 μg/ mL | [79] |
| | | *F. equiseti* AGR12 | the stem of *Rhizophora stylosa* Griff | remarkable antimicrobial activities against *B. subtilis* and *S. aureus*, with MIC value of 32 μg/mL | [16] |
| | | *F. equiseti* SCSIO 41019 | Sponge (*Callyspongia* sp.), China | strongest antibacterial activities against *S. aureus* and MRSA with MIC values of 2.0 and 3.9 μg/mL, respectively | [15] |
| **131** | fusarithioamide A | *F. chlamydosporium* | the inner tissue of *A. garcinii* leaves | antibacterial potential towards *B. cereus*, *S. aureus*, and *E. coli* with inhibition zone diameters (IZDs) of 19.0, 14.1, and 22.7 mm, respectively and MICs values of 3.1, 4.4, and 6.9 μg/mL, respectively; the most potent antifungal activity towards *C. albicans* (IZD 16.2 mm) comparable to clotrimazole (IZD 18.5 mm, positive control) | [80] |
| **132** | fusarithioamide B | *F. chlamydosporium* | *Anvillea garcinii* (Burm.f.) DC. (Asteraceae), Egypt | selective antifungal activity towards *C. albicans* (MIC 1.9 μg/mL and IZD 14.5 mm), comparing to clotrimazole (MIC 2.8 μg/mL and IZD 17.9 mm); possessed high antibacterial potential towards *E. coli*, *B. cereus*, and *S. aureus* compared to ciprofloxacin. | [81] |
| **133** | beauvericin | *F. redolens* Dzf2 | rhizomes of *Dioscorea zingiberensis* | strong activity in vitro against *E. coli*, *A. tumefaciens*, *P. lachrymans*, *X. vesicatoria*, *B. subtilis* and *S. haemolyticus* | [82] |
| | | *F. proliferatum* CECT 20569 | CECT Valencia, Spain | activity against *C. perfringens* CECT 4647, *S. enterica* | [83] |
| | | *F. oxysporum* | the bark of *C. kanehirae*, | anti-bacterial activity towards MRSA and *B. subtilis* (MIC= 3.125 | [17] |

| | | | | | |
|---|---|---|---|---|---|
| | | | Taiwan Province | μg/mL). | |
| | | *Fusarium* sp. TP-G1 | the Root of *Dendrobium officinale* Kimura et Migo | good antibacterial activities against *S. aureus* and MRSA with MIC value of 4.0 μg/mL | [5] |
| **134** | enniatin A, A1, B and B1 | *Fusarium* sp. MOBCOF-1 | The surface of the alga *Codium fragile*, the east coast of Scotland | activity against *S. aureus* and vancomycin resistant *enterococci* VRE788 | [84] |
| | | *F. tricinctum* | healthy rhizomes of *Aristolochia paucinervis*, Morocco | antibacterial activity against *B. subtilis, S. aureus, S. pneumoniae*, and *E. faecalis* with MIC values in the range 2−8 μg/mL | [64] |
| **135** | | *F. tricinctum* CECT 20150 | Spain | antibacterial activity against *C. perfringens* and *S. aureus* CECT 976 | [85] |
| | | *F. oxysporum* (N17B) | a grassy area in Lakselv, Norway | moderate activity against *C. albicans, C. neoformans,* and *M. intracellulare* | [56] |
| **136** | | *F. dimerum* | the plant tissue of *Magnolia x soulangeana* Soul. - Bod., Slovakia | effective against *B. subtilis* CCM 1999, *C. albicans* CCY 29391, *T. cutaneum* CCY 30510 and *C. neoformans* CCY 1716 | [86] |
| **137** | | *F. tricinctum* Salicorn 19 | The aerial parts of *S. bigelovii*, China | powerful antimicrobial activities toward *B. subtilis*, *E. aerogenes* and *M. tetragenus* with MIC values 13, 13 and 6 μM | [34] |
| | | *F. lateritium* Nees | - | strong antifungal activity against *E. armeniaca* | [87] |
| **138** | fusaramin | *Fusarium* sp. FKI-7550 | soil, Japan | growth inhibition against some Gram-positive bacteria (*S. aureus*, *B. subtilis* and *K. rhizophila*), one Gram-negative bacterium (*X. oryzae* pv. oryzae) and multidrug-sensitive *S. cerevisiae* 12geneΔ0HSR-iERG6 | [55] |
| **139** | 2-oxo-8-azatricyclo [9.3.1.13,7]-hexadeca-1(15),3(16),4,6,11,13-hexaen-10-one | *F. oxysporum* YP9B | the tomato plant root in Pazar-Rize, Turkey | a significant antimicrobial effect at concentrations of 0.8-6.3 μg/mL against bacterial and fungi. | [25] |
| **140** | (1-benzyl-2-methoxy-2-oxoethyl)-2-hydroxy-3-methylbutanoate | | | a strong antimicrobial effect at concentrations of 0.47-1.8 μg/mL against Gram-positive bacteria (*S. aureus*, *E. faecalis*, *S. mutans*, *B. cereus*, and *M. smegmatis*) and a moderate antimicrobial effect against Gram-negative bacteria and fungi at concentrations of 60 μg/mL | |
| **141** | 2,3-dihydroxypropanoic (11*Z*)-octadecenoic anhydride | | | a moderate antimicrobial effect at concentrations of 3.8-30.6 μg/mL against bacterial and fungi | |
| **142** | 2,3-dihydroxypropanoic, (9*E*,12*E*)-octadecadienoic anhydride | | | | |
| **143** | chrysophanol | *F. equiseti* | the brown alga *Padina pavonica,* the Red Sea | antibacterial and antifungal activity. w-hydroxyemodin and cordycepin were potent against *B. subtilis*, *S. aureus* and *C. albicans*. Cyclo (L-Pro-L-Val) was the most potent against *B. megaterium* while, 17-demethyl-2,11- dideoxy-rhizoxin was more active against *C. albicans*. chrysophanol was more active against *S. aureus* | [88] |
| **144** | ω-hydroxyemodin | | | | |
| **145** | 17-Demethyl-2,11-dideoxy-rhizoxin | | | | |
| **146** | perlolyrine | | | | |
| **147** | cordycepin | | | | |
| **148** | cyclo-(*L*-Ala-*L*-Leu) | | | | |
| **149** | cyclo(*L*-Pro-*L*-Val) | | | | |

Table S6. Detail information for *Fusarium*-derived antiviral SMs.

| No. | Name | Strain | Source | Bioactivity | Ref. |
|---|---|---|---|---|---|
| **64** | fusapyridon A | *Fusarium* sp. CPCC 400857 | the stem of tea plant | antiviral activity against the coronavirus (HCoV-OC43) with IC50 values of 13.33 and 6.65 μM, respectively | [89] |
| **99** | fusaricide | *Fusarium* sp. | Flowers of sourwood *(Oxydendron arboreum),* Georgia State Botanical Garden in Athens | anti-HIV | [52] |
| **105** | oxysporidinone | *Fusarium* sp. CPCC 400857 | the stem of tea plant | antiviral activity against the coronavirus (HCoV-OC43) with IC50 values of 13.33 and 6.65 μM, respectively | [89] |
| **135** | enniatin A1 | *Fusarium* sp. | - | The compounds protected human lymphoblastoid cells from HIV-1 induced cell killing with an in vitro"therapeutic index" of approximately 200 ($IC_{50}$ = 1.9 μg $ml^{-1}$, $EC_{50}$ = 0.01 μg $ml^{-1}$) | [90] |
| **136** | enniatin B | | | | |
| **137** | enniatin B1 | | | | |
| **140** | (1-benzyl-2-methoxy-2-oxoethyl)-2-hydroxy-3-methylbutanoate | *F. oxysporum* YP9B | the tomato plant root in Pazar-Rize, Turkey | antiviral activity against HSV type-1 was determined to be 0.312 μM | [25] |
| **141** | 2,3-dihydroxypropanoic (11*Z*)- octadecenoic anhydride | *F. oxysporum* YP9B | the tomato plant root in Pazar-Rize, Turkey | antiviral activity against HSV type-1 was determined to be 1.25 μM | [25] |
| **142** | 2,3-dihydroxypropanoic, (9*E*,12*E*)-octadecadienoic anhydride | | | | |
| **144** | ω-hydroxyemodin | *F. equiseti* | the brown alga *Padina Pavonica,* the Red Sea | Cordycepin showed less potency, 17-demethyl-2,11-dideoxy-rhizoxin and perlolyrine were moderately potent. Cyclo (*L*-Pro-*L*-Val) showed good potency against HCV NS3/4A protease while, ω- hydroxyemodin was the most potent HCVPR inhibitors | [88] |
| **145** | 17-Demethyl-2,11-dideoxy-rhizoxin | | | | |
| **146** | perlolyrine | | | | |
| **147** | cordycepin | | | | |
| **149** | cyclo(*L*-Pro-*L*-Val) | | | | |
| **150** | fusaindoterpene B | *Fusarium sp.* L1 | the inner tissue of the sea star *Acanthaster planci*, China | inhibitory activity against the Zika virus (ZIKV) in a standard plaque assay with $EC_{50}$ values of 7.5, 4.2, and 5.0 μM, respectively. | [91] |
| **151** | JBIR-03 | | | | |
| **152** | 1,2-bis(1*H*-indol-3-yl) ethane-1,2-dione | | | | |
| **153** | ara-A | *F. equiseti* | the brown alga *Padina Pavonica*, the Red Sea | ara-A showed less potency, cyclic tetrapeptidecyclo-[Phenylalanyl-proleu-pro], 5-chloro-3,6-dihydroxy-2-methyl-1,4-benzoquinone and were moderately potent; griseoxanthone C showed good potency against HCV NS3/4A protease while, and cyclo (*L*-Tyr-*L*-Pro) was the most potent HCVPR inhibitors | [88] |
| **154** | cyclic tetrapeptidecyclo-[Phenylalanyl-proleu-pro] | | | | |
| **155** | cyclo (*L*-Tyr-*L*-Pro) | | | | |
| **156** | 5-chloro-3,6-dihydroxy-2-methyl-1,4-benzoquinone | | | | |
| **157** | griseoxanthone C | | | | |
| **158** | coculnol | coculture of *F. solani* FKI-6853 and *Talaromyces* sp. FKA-65 | soil, Japan | an inhibitory effect (with $IC_{50}$ value of 283 μg $ml^{-1}$) against A/PR/8/34 (H1N1) | [92] |

Table S7. Detail information for *Fusarium*-derived antiparasitic SMs.

| No. | Name | Strain | Source | Bioactivity | Ref. |
|---|---|---|---|---|---|
| **23** | anhydrofusarubin | *Fusarium* sp.PSU-F135 | marine brown alga *Colpomenia sinuosa* | weak antimalarial (*P. falciparum* K1) activity, with $IC_{50}$ values in the range 9.8-14 μM. | [12] |
| **28** | 9α-hydroxyhalorosellinia A | *Fusarium* sp. PSU-F14 | a gorgonian sea fan | | |
| **29** | nigrosporin B | | | | |
| **59** | NG-391 | *Fusarium* sp. RK97-94 | - | antimalarial activity ($IC_{50}$ = 1.8 μM) (*P. falciparum* 3D7) | [93] |
| **104** | fusaripeptide A | *Fusarium* sp. | the roots of *Mentha longifolia* L. (Labiatae), Saudi Arabia | significant antiplasmodial activity toward *P. falciparum* (D6 clone) with an $IC_{50}$ value of 0.34 μM | [49] |
| **109** | javanicin | *Fusarium* sp. PSU-F135 | marine brown alga *Colpomenia sinuosa* | weak antimalarial (*P. falciparum* K1) activity, with $IC_{50}$ values in the range 9.8-14 μM. | [12] |
| **117** | bikaverin | *F. fujikuroi* | - | specifically effective against *Leishmania brasiliensis* | [94] |
| **133** | beauvericin | *Fusarium sp.* WC9 | *Caesalpinia echinata* Lam. (Brazilwood), Brazil | inhibit *T. cruzi* with $IC_{50}$ 2.43 μM | [95] |
| | | *F. oxysporum* SS46 | medicinal plant *Smallanthus sonchifolius* (Poepp.) H. Rob. | promising activity against *Leishmania braziliensis* | [96] |
| **134** | enniatin A | *F. tricinctum* Corda | the fruits of *Hordeum sativum* Jess | mild antileishmanial activities (displayed inhibition of the activity of thioredoxin reductase enzyme of *Plasmodium falciparum*) | [3] |
| **135** | enniatin A1 | | | | |
| **136** | enniatin B | | | | |
| **137** | enniatin B1 | | | | |
| **159** | bostrycin | *Fusarium* sp. PSU-F14 | a gorgonian sea fan | weak antimalarial (*P. falciparum* K1) activity, with $IC_{50}$ values in the range 9.8-14 μM. | [12] |
| **160** | solaninaphthoquione | *F. solani* PSURSPG227 | Soil, Thailand | weak antimalarial (*P. falciparum* K1) activity ($IC_{50}$ of 26.1 μM) | [97] |
| **161** | integracides F | *Fusarium* sp. | the roots of *Mentha longifolia* L., Saudi Arabia | significant anti-leishmanial activity towards *Leishmania donovani* with $IC_{50}$ values of 3.74 and 2.53 μg/mL, respectively and $IC_{90}$ values of 5.11 and 8.89 μg/mL, respectively. | [98] |
| **162** | integracides G | | | | |
| **163** | integracides H | | | Significant anti-leishmanial activity towards *Leishmania donovani* with $IC_{50}$ values of 4.75 and 3.29 μM, respectively compared to pentamidine ($IC_{50}$ 6.35 μM). | [99] |
| **164** | integracides J | | | | |
| **165** | dihydroNG391 | *Fusarium* sp. RK97-94 | - | antimalarial activity ($IC_{50}$ = 62.1 μM) (*P. falciparum* 3D7) | [93,100] |
| **166** | dihydrolucilactaene | | | potent antimalarial activity ($IC_{50}$ = 0.0015 μM) (*P. falciparum* 3D7 and K1) | |
| **167** | lucilactaene | | | potent antimalarial activity ($IC_{50}$ = 0.15 μM) (*P. falciparum* 3D7) | |
| **168** | 13α-hydroxylucilactaene | | | potent antimalarial activity ($IC_{50}$ = 0.68 μM) (*P. falciparum* 3D7) | |
| **169** | demethyllucilactaene | | | antimalarial activity ($IC_{50}$ = 43.9 μM) (*P. falciparum* 3D7) | |
| **170** | (8*Z*)-demethyllucilactaene | | | weak antimalarial activity (*P. falciparum* 3D7) | |
| **171** | prelucilactaene G | | | antimalarial activity ($IC_{50}$ = 13.3 μM) (*P. falciparum* 3D7) | |

| | | | | | |
|---|---|---|---|---|---|
| **172** | prelucilactaene H | | | antimalarial activity ($IC_{50}$ = 15.6 µM) (*P. falciparum* 3D7) | [101] |
| **173** | prelucilactaene A | | | antimalarial activity ($IC_{50}$ = 15.2 µM) (*P. falciparum* 3D7) | |
| **174** | prelucilactaene B | | | antimalarial activity ($IC_{50}$ = 26.9 µM) (*P. falciparum* 3D7) | |
| **175** | prelucilactaene E | | | antimalarial activity ($IC_{50}$ = 3.5 µM) (*P. falciparum* 3D7) | |
| **176** | prelucilactaene F | | | antimalarial activity ($IC_{50}$ = 4.3 µM) (*P. falciparum* 3D7) | |
| **177** | apicidin | *F. pallidoroseum*<br>*F. fujikuroi* | - | antimalarial activity (*Plasmodium berghei*) | [102]<br>[103]<br>[104] |
| **178** | apicidin A | | | | |
| **179** | apicidin B | | | | |
| **180** | apicidin C | | | | |
| **181** | apicidin $D_1$ | | | | |
| **182** | apicidin $D_2$ | | | | |
| **183** | apicidin $D_3$ | | | | |
| **184** | apicidin E | | | | |
| **185** | apicidin F | | | The determined $IC_{50}$ value of 0.67 µM (average of two replicates) is about 3-fold higher compared to the IC50 value of apicidin, which is about 0.2 µM. | [105] |

## References


1. Chen, J.; Bai, X.; Hua, Y.; Zhang, H.; Wang, H. Fusariumins C and D, two novel antimicrobial agents from *Fusarium oxysporum* ZZP-R1 symbiotic on Rumex madaio Makino. *Fitoterapia* **2019**, *134*, 1-4, doi:10.1016/j.fitote.2019.01.016.
2. Brill, G.M.; Kati, W.M.; Montgomery, D.; Karwowski, J.P.; Humphrey, P.E.; Jackson, M.; Clement, J.J.; Kadam, S.; Chen, R.H.; McAlpine, J.B. Novel triterpene sulfates from *Fusarium compactum* using a rhinovirus 3C protease inhibitor screen. *J. Antibiot (Tokyo).* **1996**, *49*, 541-546, doi:10.7164/antibiotics.49.541.
3. Zaher, A.M.; Makboul, M.A.; Moharram, A.M.; Tekwani, B.L.; Calderon, A.I. A new enniatin antibiotic from the endophyte *Fusarium tricinctum* Corda. *J. Antibiot (Tokyo).* **2015**, *68*, 197-200, doi:10.1038/ja.2014.129.
4. Tomoda, H.; Nishida, H.; Huang, X.-H.; Masuma, R.; Kim, Y.K.; Omura, S. New cyclodepsipeptides, enniatins D, E and f produced by *Fusarium* sp. FO-1305Áü. *J. Antibiot (Tokyo).* **1992**, *45*, 1207-1215.
5. Shi, S.; Li, Y.; Ming, Y.; Li, C.; Li, Z.; Chen, i.; Luo, M. Biological activity and chemical composition of the endophytic fungus *Fusarium* sp. TP-G1 obtained from the root of *Dendrobium officinale* Kimura et Migo. *Rec. Nat. Prod.* **2018**, *12*, 549-556, doi:10.25135/rnp.62.17.12.201.
6. Inokoshi, J.; Shigeta, N.; Fukuda, T.; Uchida, R.; Nonaka, K.; Masuma, R.; Tomoda, H. *Epi*-trichosetin, a new undecaprenyl pyrophosphate synthase inhibitor, produced by *Fusarium oxysporum* FKI-4553. *J. Antibiot (Tokyo).* **2013**, *66*, 549-554, doi:10.1038/ja.2013.44.
7. Du, Z.; Song, C.; Yu, B.; Luo, X. Secondary metabolites produced by *Fusarium* sp. 2TnP1-2, an endophytic fungus from *Trewia nudiflora*. *Chin. J. Med. Chem.* **2008**, *18*, 452-456.
8. Nenkep, V.; Yun, K.; Son, B.W. Oxysporizoline, an antibacterial polycyclic quinazoline alkaloid from the marine-mudflat-derived fungus *Fusarium oxysporum*. *J. Antibiot (Tokyo).* **2016**, *69*, 709-711, doi:10.1038/ja.2015.137.
9. Nenkep, V.; Yun, K.; Zhang, D.; Choi, H.D.; Kang, J.S.; Son, B.W. Induced production of bromomethylchlamydosporols A and B from the marine-derived fungus *Fusarium tricinctum*. *J. Nat. Prod.* **2010**, *73*, 2061-2063, doi:10.1021/np1005289.
10. Alfattani, A.; Marcourt, L.; Hofstetter, V.; Queiroz, E.F.; Leoni, S.; Allard, P.M.; Gindro, K.; Stien, D.; Perron, K.; Wolfender, J.L. Combination of pseudo-LC-NMR and HRMS/MS-based molecular networking for the rapid identification of mntimicrobial metabolites From *Fusarium petroliphilum*. *Front. Mol. Biosci.* **2021**, *8*, 725691-725715, doi:10.3389/fmolb.2021.725691.
11. Baker, R.A.; Tatum, J.H.; Nemec Jr, S. Antimicrobial activity of naphthoquinones from Fusaria. *Mycopathologia* **1990**, *111*, 9-15, doi:10.1007/bf02277294.
12. Trisuwan, K.; Khamthong, N.; Rukachaisirikul, V.; Phongpaichit, S.; Preedanon, S.; Sakayaroj, J. Anthraquinone, cyclopentanone, and naphthoquinone derivatives from the sea fan-derived fungi *Fusarium* spp. PSU-F14 and PSU-F135. *J. Nat. Prod.* **2010**, *73*, 1507-1511, doi:10.1021/np100282k.
13. Kornsakulkarn, J.; Dolsophon, K.; Boonyuen, N.; Boonruangprapa, T.; Rachtawee, P.; Prabpai, S.; Kongsaeree, P.; Thongpanchang, C. Dihydronaphthalenones from endophytic fungus *Fusarium* sp. BCC14842. *Tetrahedron* **2011**, *67*, 7540-7547, doi:10.1016/j.tet.2011.07.078.

14. Shah, A.; Rather, M.A.; Hassan, Q.P.; Aga, M.A.; Mushtaq, S.; Shah, A.M.; Hussain, A.; Baba, S.A.; Ahmad, Z. Discovery of anti-microbial and anti-tubercular molecules from *Fusarium solani*: an endophyte of *Glycyrrhiza glabra*. *J. Appl. Microbiol.* **2017**, *122*, 1168-1176, doi:10.1111/jam.13410.
15. Chen, C.; Luo, X.; Li, K.; Guo, C.; Li, J.; Lin, X. Antibacterial secondary metabolites from a marine sponge-derived fungus *Fusarium equiseti* SCSIO 41019. *Chin. J. Antibiot.* **2019**, *44*, 1035-1040, doi:10.13461/j.cnki.cja.006629.
16. Wang, J.; Lu, W.; Min, C. The endophytic fungus AGR12 in the stem of *Rhizophora stylosa* Griff and its antibacterial metabolites. *Chin. J. Antibiot.* **2011**, *36*, 102-106.
17. Wang, Q.X.; Li, S.F.; Zhao, F.; Dai, H.Q.; Bao, L.; Ding, R.; Gao, H.; Zhang, L.X.; Wen, H.A.; Liu, H.W. Chemical constituents from endophytic fungus *Fusarium oxysporum*. *Fitoterapia* **2011**, *82*, 777-781, doi:10.1016/j.fitote.2011.04.002.
18. Mohamed, G.A.; Ibrahim, S.R.M.; Alhakamy, N.A.; Aljohani, O.S. Fusaroxazin, a novel cytotoxic and antimicrobial xanthone derivative from *Fusarium oxysporum*. *Nat. Prod. Res.* **2022**, *36*, 952-960, doi:10.1080/14786419.2020.1855165.
19. Renner, M.K.; Jensen, P.R.; Fenical, W. Neomangicols: structures and absolute stereochemistries of unprecedented halogenated sesterterpenes from a marine fungus of the genus *Fusarium*. *J. Org. Chem.* **1998**, *63*, 8346-8354, doi:10.1021/jo981226b.
20. Li, S.; Shao, M.-W.; Lu, Y.-H.; Kong, L.-C.; Jiang, D.-H.; Zhang, Y.-L. Phytotoxic and antibacterial metabolites from *Fusarium proliferatum* ZS07 isolated from the gut of Long-horned Grasshoppers. *J. Agric. Food Chem.* **2014**, *62*, 8997-9001, doi:10.1021/jf502484n.
21. Jiang, C.X.; Li, J.; Zhang, J.M.; Jin, X.J.; Yu, B.; Fang, J.G.; Wu, Q.X. Isolation, identification, and activity evaluation of chemical constituents from soil fungus *Fusarium avenaceum* SF-1502 and endophytic fungus *Fusarium proliferatum* AF-04. *J. Agric. Food Chem.* **2019**, *67*, 1839-1846, doi:10.1021/acs.jafc.8b05576.
22. Hussain, H.; Drogies, K.-H.; Al-Harrasi, A.; Hassan, Z.; Shah, A.; Rana, U.A.; Green, I.R.; Draeger, S.; Schulz, B.; Krohn, K. Antimicrobial constituents from endophytic fungus *Fusarium* sp. *Asian Pac. J. Trop. Dis.* **2015**, *5*, 186-189, doi:10.1016/s2222-1808(14)60650-2.
23. Lu, W.; Zhu, G.; Yuan, W.; Han, Z.; Dai, H.; Basiony, M.; Zhang, L.; Liu, X.; Hsiang, T.; Zhang, J. Two novel aliphatic unsaturated alcohols isolated from a pathogenic fungus *Fusarium proliferatum*. *Synth. Syst. Biotechnol.* **2021**, *6*, 446-451, doi:10.1016/j.synbio.2021.10.001.
24. Wen, H.; Li, Y.; Liu, X.; Ye, W.; Yao, X.; Che, Y. Fusagerins A-F, new alkaloids from the fungus *Fusarium* sp. *Nat. Prod. Bioprospect.* **2015**, *5*, 195-203, doi:10.1007/s13659-015-0067-1.
25. Kılıç, G.; Tosun, G.; Bozdeveci, A.; Erik, İ.; Öztürk, E.; Reis, R.; Sipahi, H.; Cora, M.; Karaoğlu, Ş.A.; Yaylı, N. Antimicrobial, cytotoxic, antiviral effects, and apectroscopic characterization of metabolites produced by *fusarium oxysporum* YP9B. *Rec. Nat. Prod.* **2021**, *15*, 547-567, doi:10.25135/rnp.208.20.06.1674.
26. Valla, A.; Giraud, M.; Labia, R.; Morand, A. In vitro inhibitory activity against bacteria of a fusarium mycotoxin and new synthetic derivatives. *Bull. Soc. Chim. Fr.* **1997**, *6*, 601-603.
27. Kyekyeku, J.O.; Kusari, S.; Adosraku, R.K.; Bullach, A.; Golz, C.; Strohmann, C.; Spiteller, M. Antibacterial secondary metabolites from an endophytic fungus, *Fusarium solani* JK10. *Fitoterapia* **2017**, *119*, 108-114, doi:10.1016/j.fitote.2017.04.007.

28. Sibero, M.T.; Zhou, T.; Fukaya, K.; Urabe, D.; Radjasa, O.K.K.; Sabdono, A.; Trianto, A.; Igarashi, Y. Two new aromatic polyketides from a sponge-derived *Fusarium*. *Beilstein. J. Org. Chem.* **2019**, *15*, 2941-2947, doi:10.3762/bjoc.15.289.
29. Tsuchinari, M.; Shimanuki, K.; Hiramatsu, F.; Murayama, T.; Koseki, T.; Shiono, Y. Fusapyridons A and B, novel pyridone alkaloids from an endophytic fungus, *Fusarium* sp. YG-45. *Z. Naturforsch. B.* **2007**, *62*, 1203-1207, doi:10.1515/znb-2007-0916.
30. Supratman, U.; Hirai, N.; Sato, S.; Watanabe, K.; Malik, A.; Annas, S.; Harneti, D.; Maharani, R.; Koseki, T.; Shiono, Y. New naphthoquinone derivatives from *Fusarium napiforme* of a mangrove plant. *Nat. Prod. Res.* **2021**, *35*, 1406-1412, doi:10.1080/14786419.2019.1650358.
31. Khan, N.; Afroz, F.; Begum, M.N.; Roy Rony, S.; Sharmin, S.; Moni, F.; Mahmood Hasan, C.; Shaha, K.; Sohrab, M.H. Endophytic *Fusarium solani*: A rich source of cytotoxic and antimicrobial napthaquinone and aza-anthraquinone derivatives. *Toxicol. Rep.* **2018**, *5*, 970-976, doi:10.1016/j.toxrep.2018.08.016.
32. Haraguchi, H.; Yokoyama, K.; Oike, S.; Ito, M.; Nozaki, H. Respiratory stimulation and generation of superoxide radicals in *Pseudomonas aeruginosa* by fungal naphthoquinones. *Arch. Microbiol.* **1997**, *167*, 6-10, doi:10.1007/s002030050409.
33. Yan, C.; Liu, W.; Li, J.; Deng, Y.; Chen, S.; Liu, H. Bioactive terpenoids from Santalum album derived endophytic fungus *Fusarium* sp. YD-2. *RSC. Adv.* **2018**, *8*, 14823-14828, doi:10.1039/c8ra02430h.
34. Zhang, J.; Liu, D.; Wang, H.; Liu, T.; Xin, Z. Fusartricin, a sesquiterpenoid ether produced by an endophytic fungus *Fusarium tricinctum* Salicorn 19. *Eur. Food Res. Technol.* **2014**, *240*, 805-814, doi:10.1007/s00217-014-2386-6.
35. Dong, J.W.; Cai, L.; Li, X.J.; Duan, R.T.; Shu, Y.; Chen, F.Y.; Wang, J.P.; Zhou, H.; Ding, Z.T. Production of a new tetracyclic triterpene sulfate metabolite sambacide by solid-state cultivated *Fusarium sambucinum* B10.2 using potato as substrate. *Bioresour. Technol.* **2016**, *218*, 1266-1270, doi:10.1016/j.biortech.2016.07.014.
36. Shu, R.; Wang, F.; Yang, Y.; Liu, Y.; Tan, R. Antibacterial and xanthine oxidase inhibitory cerebrosides from *Fusarium* sp. IFB-121, and endophytic fungus in Quercus variabilis. *Lipids* **2004**, *39*, 667-673, doi:10.1007/s11745-004-1280-9.
37. Sebastià, N.; Meca, G.; Soriano, J.M.; Mañes, J. Antibacterial effects of enniatins J(1) and J(3) on pathogenic and lactic acid bacteria. *Food Chem. Toxicol.* **2011**, *49*, 2710-2717, doi:10.1016/j.fct.2011.06.070.
38. Chen, C.; Imamura, N.; Nishijima, M.; Adachi, K.; Sakai, M.; Sano, H. Halymecins, new antimicroalgal substances produced by fungi isolated from marine algae. *J. Antibiot (Tokyo).* **1996**, *49*, 998-1005, doi:10.7164/antibiotics.49.998.
39. Shiono, Y.; Shibuya, F.; Murayama, T.; Koseki, T.; Poumale, H.M.P.; Ngadjui, B.T. A polyketide metabolite from an endophytic *Fusarium equiseti* in a medicinal plant. *Z. Naturforsch. B.* **2013**, *68*, 289-292, doi:10.5560/znb.2013-3014.
40. Jackson, M.; Frost, D.J.; Karwowski, J.P.; Humphrey, P.E.; Dahod, S.K.; Choi, W.S.; Brandt, K.; Malmberg, L.-H.; Rasmussen, R.R.; Scherr, M.H. Fusacandins A and B; novel antifungal antibiotics of the papulacandin class from *Fusarium sambucinum J. Antibiot (Tokyo).* **1995**, *48*, 608-613, doi:10.7164/antibiotics.48.608.

41. Chen, R.H.; Tennant, S.; Frost, D.; O'beirne, M.J.; Karwowski, J.P.; Humphrey, P.E.; Malmberg, L.-H.; Choi, W.; Brandt, K.D.; West, P. Discovery of saricandin, a novel papulacandin, from a *Fusarium* species. *J. Antibiot (Tokyo).* **1996**, *49*, 596-598, doi:10.7164/antibiotics.49.596.
42. Brady, S.F.; Clardy, J. CR377, a new pentaketide antifungal agent isolated from an endophytic fungus. *J. Nat. Prod.* **2000**, *63*, 1447-1448, doi:10.1021/np990568p.
43. Arunpanichlert, J.; Rukachaisirikul, V.; Sukpondma, Y.; Phongpaichit, S.; Supaphon, O.; Sakayaroj, J. A *β*-resorcylic macrolide from the seagrass-derived fungus *Fusarium* sp. PSU-ES73. *Arch. Pharm. Res.* **2011**, *34*, 1633-1637, doi:10.1007/s12272-011-1007-1.
44. Saetang, P.; Rukachaisirikul, V.; Phongpaichit, S.; Sakayaroj, J.; Shi, X.; Chen, J.; Shen, X. *β*-Resorcylic macrolide and octahydronaphthalene derivatives from a seagrass-derived fungus *Fusarium* sp. PSU-ES123. *Tetrahedron* **2016**, *72*, 6421-6427, doi:10.1016/j.tet.2016.08.048.
45. Hiramatsu, F.; Miyajima, T.; Murayama, T.; Takahashi, K.; Koseki, T.; Shiono, Y. Isolation and structure elucidation of neofusapyrone from a marine-derived *Fusarium* species, and structural revision of fusapyrone and deoxyfusapyrone. *J. Antibiot (Tokyo).* **2006**, *59*, 704-709, doi:10.1038/ja.2006.94.
46. Xu, X.; Zhao, S.; Yu, Y.; Chen, Z.; Shen, H.; Zhou, L. Beauvericin K, a new antifungal beauvericin analogue from a marine-derived *Fusarium* sp. *Nat. Prod. Commun.* **2016**, *11*, 1825–1826, doi:10.1177/1934578x1601101213.
47. Baráth, Z.; Baráthová, H.; Betina, V.; Nemec, P. Ramihyphins—Antifungal and morphogenic antibiotics from *Fusarium* sp. S-435. *Folia. Microbiol.* **1974**, *19*, 507-511, doi:10.1007/bf02872917.
48. Sawai, K.; Okuno, T.; Terada, Y.; Harada, Y.; Sawamura, K.; Sasaki, H.; Takao, S. Isolation and properties of two antifungal substances from *Fusarium solani*. *Agri. Biol. Chem.* **2014**, *45*, 1223-1228, doi:10.1080/00021369.1981.10864674.
49. Ibrahim, S.R.M.; Abdallah, H.M.; Elkhayat, E.S.; Al Musayeib, N.M.; Asfour, H.Z.; Zayed, M.F.; Mohamed, G.A. Fusaripeptide A: new antifungal and anti-malarial cyclodepsipeptide from the endophytic fungus *Fusarium* sp. *J. Asian. Nat. Prod. Res.* **2018**, *20*, 75-85, doi:10.1080/10286020.2017.1320989.
50. Overy, D.; Calati, K.; Kahn, J.N.; Hsu, M.J.; Martin, J.; Collado, J.; Roemer, T.; Harris, G.; Parish, C.A. Isolation and structure elucidation of parnafungins C and D, isoxazolidinone-containing antifungal natural products. *Bioorg. Med. Chem. Lett.* **2009**, *19*, 1224-1227, doi:10.1016/j.bmcl.2008.12.081.
51. Parish, C.A.; Smith, S.K.; Calati, K.; Zink, D.; Wilson, K.; Roemer, T.; Jiang, B.; Xu, D.; Bills, G.; Platas, G. Isolation and structure elucidation of parnafungins, antifungal natural products that inhibit mRNA polyadenylation. *J. Am. Chem. Soc.* **2008**, *130*, 7060-7066, doi:10.1021/ja711209p.
52. McBrien, K.D.; Gao, Q.; Huang, S.; Klohr, S.E.; Wang, R.R.; Pirnik, D.M.; Neddermann, K.M.; Bursuker, I.; Kadow, K.F.; Leet, J.E. Fusaricide, a new cytotoxic *N*-hydroxypyridone from *Fusarium* sp. *J. Nat. Prod.* **1996**, *59*, 1151-1153, doi:10.1021/np960521t.
53. Hilário, F.; Chapla, V.; Araujo, A.; Sano, P.; Bauab, T.; dos Santos, L. Antimicrobial screening of endophytic fungi isolated from the aerial parts of *Paepalanthus chiquitensis* (Eriocaulaceae) led to the isolation of secondary metabolites produced by *Fusarium fujikuroi*. *J. Braz. Chem. Soc.* **2016**, *28*, 1389-1395, doi:10.21577/0103-5053.20160312.
54. M. Ibrahim, S.; Mohamed, G.; Khayat, M.; Al Haidari, R.; El-Kholy, A.; Zayed, M. A new antifungal aminobenzamide derivative from the endophytic fungus *Fusarium* sp. *Pharmacogn.*

*Mag.* **2019**, *15*, 204-207, doi:10.4103/pm.pm_476_18.

55. Sakai, K.; Unten, Y.; Iwatsuki, M.; Matsuo, H.; Fukasawa, W.; Hirose, T.; Chinen, T.; Nonaka, K.; Nakashima, T.; Sunazuka, T.; et al. Fusaramin, an antimitochondrial compound produced by *Fusarium* sp., discovered using multidrug-sensitive *Saccharomyces cerevisiae*. *J. Antibiot (Tokyo).* **2019**, *72*, 645-652, doi:10.1038/s41429-019-0197-5.
56. Jayasinghe, L.; Abbas, H.K.; Jacob, M.R.; Herath, W.H.; Nanayakkara, N.D. *N*-Methyl-4-hydroxy-2-pyridinone analogues from *Fusarium oxysporum*. *J. Nat. Prod.* **2006**, *69*, 439-442.
57. Strongman, D.; Miller, J.; Calhoun, L.; Findlay, J.; Whitney, N. The biochemical basis for interference competition among some lignicolous marine fungi. *Bot. Mar.* **1987**, *30*, 21-26, doi:10.1515/botm.1987.30.1.21.
58. Pedersen, P.B.; Miller, J.D. The fungal metabolite culmorin and related compounds. *Nat. Toxins* **1999**, *7*, 305-309, doi:10.1002/1522-7189(199911/12)7:6<305::aid-nt72>3.0.co;2-g.
59. Arnstein, H.; Cook, A. Javanicin. An antibacterial pigment from *Fusarium javanicum*. *J. Chem. Soc.* **1947**, 1021-1028.
60. Kurobane, I.; Zaita, N.; Fukuda, a. New metabolites of *Fusarium martii* related to dihydrofusarubin. *J. Antibiot (Tokyo).* **1986**, *39*, 205-214, doi:10.7164/antibiotics.39.205.
61. Deshmukh, R.; Mathew, A.; Purohit, H.J. Characterization of antibacterial activity of bikaverin from *Fusarium* sp. HKF15. *J. Biosci. Bioeng.* **2014**, *117*, 443-448, doi:10.1016/j.jbiosc.2013.09.017.
62. Limón, M.C.; Rodríguez-Ortiz, R.; Avalos, J. Bikaverin production and applications. *Appl. Microbiol. Biotechnol.* **2010**, *87*, 21-29, doi:10.1007/s00253-010-2551-1.
63. Ariantari, N.P.; Frank, M.; Gao, Y.; Stuhldreier, F.; Kiffe-Delf, A.-L.; Hartmann, R.; Höfert, S.-P.; Janiak, C.; Wesselborg, S.; Müller, W.E.G.; et al. Fusaristatins D–F and (7S,8R)-(−)-chlamydospordiol from *Fusarium* sp. BZCB-CA, an endophyte of *Bothriospermum chinense*. *Tetrahedron* **2021**, *85*, 132065-132071, doi:10.1016/j.tet.2021.132065.
64. Ola, A.R.B.; Thomy, D.; Lai, D.; Brötz-Oesterhelt, H.; Proksch, P. Inducing secondary metabolite production by the endophytic fungus *Fusarium tricinctum* through coculture with *Bacillus subtilis*. *J. Nat. Prod.* **2013**, *76*, 2094-2099, doi:10.1021/np400589h.
65. Bushnell, G.W.; Li, Y.-L.; Poulton, G.A. Pyrones. X. Lateropyrone, a new antibiotic from the fungus *Fusarium lateritium* Nees. *Can. J. Chem.* **1984**, *62*, 2101-2106, doi:10.1139/v84-358.
66. Clark, T.N.; Carroll, M.; Ellsworth, K.; Guerrette, R.; Robichaud, G.A.; Johnson, J.A.; Gray, C.A. Antibiotic mycotoxins from an endophytic *Fusarium acuminatum* isolated from the medicinal plant *Geum macrophyllum*. *Nat. Prod. Commun.* **2018**, *13*, 1934578X1801301017, doi:10.1177/1934578x1801301017.
67. Okada, H.; Nagashima, M.; Suzuki, H.; Nakajima, S.; Kojiri, K.; Suda, H. BE-29602, a new member of the papulacandin family. *J. Antibiot (Tokyo).* **1996**, *49*, 103-106, doi:10.7164/antibiotics.49.103.
68. Kobayashi, H.; Sunaga, R.; Furihata, K.; Morisaki, N.; IWasaki, S. Isolation and structures of an antifungal antibiotic, fusarielin A, and related compounds produced by a *Fusarium* sp. *J. Antibiot (Tokyo).* **1995**, *48*, 42-52, doi:10.7164/antibiotics.48.42.
69. Liang, X.A.; Ma, Y.M.; Zhang, H.C.; Liu, R. A new helvolic acid derivative from an endophytic *Fusarium* sp. of Ficus carica. *Nat. Prod. Res.* **2016**, *30*, 2407-2412, doi:10.1080/14786419.2016.1190722.

70. Janevska, S.; Arndt, B.; Niehaus, E.-M.; Burkhardt, I.; Rösler, S.M.; Brock, N.L.; Humpf, H.-U.; Dickschat, J.S.; Tudzynski, B. Gibepyrone biosynthesis in the rice pathogen *Fusarium fujikuroi* is facilitated by a small polyketide synthase gene cluster. *J. Biol. Chem.* **2016**, *291*, 27403-27420, doi:10.1074/jbc.m116.753053.
71. Zhou, G.; Qiao, L.; Zhang, X.; Sun, C.; Che, Q.; Zhang, G.; Zhu, T.; Gu, Q.; Li, D. Fusaricates H-K and fusolanones A-B from a mangrove endophytic fungus *Fusarium solani* HDN15-410. *Phytochemistry* **2019**, *158*, 13-19, doi:10.1016/j.phytochem.2018.10.035.
72. Evidente, A.; Conti, L.; Altomare, C.; Bottalico, A.; Sindona, G.; Segre, A.L.; Logrieco, A. Fusapyrone and deoxyfusapyrone, two antifungal α-pyrones from *Fusarium semitectum*. *Nat. Toxins* **1994**, *2*, 4-13, doi:10.1002/nt.2620020103.
73. Altomare, C.; Perrone, G.; Zonno, M.C.; Evidente, A.; Pengue, R.; Fanti, F.; Polonelli, L. Biological characterization of fusapyrone and deoxyfusapyrone, two bioactive secondary metabolites of *Fusarium semitectum*. *J. Nat. Prod.* **2000**, *63*, 1131-1135.
74. Son, S.; Kim, H.; Choi, G.; Lim, H.; Jang, K.; Lee, S.; Lee, S.; Sung, N.; Kim, J.C. Bikaverin and fusaric acid from *Fusarium oxysporum* show antioomycete activity against *Phytophthora infestans*. *J. Appl. Microbiol.* **2008**, *104*, 692-698, doi:10.1111/j.1365-2672.2007.03581.x.
75. Bacon, C.W.; Hinton, D.M.; Hinton, A., Jr. Growth-inhibiting effects of concentrations of fusaric acid on the growth of *Bacillus mojavensis* and other biocontrol *Bacillus* species. *J. Appl. Microbiol.* **2006**, *100*, 185-194, doi:10.1111/j.1365-2672.2005.02770.x.
76. Poleto, L.; da Rosa, L.O.; Fontana, R.C.; Rodrigues, E.; Poletto, E.; Baldo, G.; Paesi, S.; Sales-Campos, C.; Camassola, M. Production of antimicrobial metabolites against pathogenic bacteria and yeasts by *Fusarium oxysporum* in submerged culture processes. *Bioproc. Biosyst. Eng.* **2021**, *44*, 1321-1332, doi:10.1007/s00449-021-02538-2.
77. Vesonder, R.F.; Tjarks, L.w.; Rohwedder, W.K.; Burmeister, H.R.; Laugal, J.A. Equisetin, an antibiotic from *Fusarium equisetin* NRRL 5537, identified as a derivative of *N*-methyl-2, 4-pyrollidone. *J. Antibiot (Tokyo).* **1979**, *32*, 759-761, doi:10.7164/antibiotics.32.759.
78. Whitt, J.; Shipley, S.M.; Newman, D.J.; Zuck, K.M. Tetramic Acid Analogues Produced by Coculture of *Saccharopolyspora erythraea* with *Fusarium pallidoroseum*. *J. Nat. Prod.* **2014**, *77*, 173-177, doi:10.1021/np400761g.
79. Ratnaweera, P.B.; de Silva, E.D.; Williams, D.E.; Andersen, R.J. Antimicrobial activities of endophytic fungi obtained from the arid zone invasive plant Opuntia dillenii and the isolation of equisetin, from endophytic *Fusarium* sp. *Bmc. Complem. Altern. M.* **2015**, *15*, 220, doi:10.1186/s12906-015-0722-4.
80. Ibrahim, S.R.M.; Elkhayat, E.S.; Mohamed, G.A.A.; Fat'hi, S.M.; Ross, S.A. Fusarithioamide A, a new antimicrobial and cytotoxic benzamide derivative from the endophytic fungus *Fusarium chlamydosporium*. *Biochem. Biophys. Res. Commun.* **2016**, *479*, 211-216, doi:10.1016/j.bbrc.2016.09.041.
81. Ibrahim, S.R.M.; Mohamed, G.A.; Al Haidari, R.A.; Zayed, M.F.; El-Kholy, A.A.; Elkhayat, E.S.; Ross, S.A. Fusarithioamide B, a new benzamide derivative from the endophytic fungus *Fusarium chlamydosporium* with potent cytotoxic and antimicrobial activities. *Bioorg. Med. Chem.* **2018**, *26*, 786-790, doi:10.1016/j.bmc.2017.12.049.
82. Xu, L.; Wang, J.; Zhao, J.; Li, P.; Shan, T.; Wang, J.; Li, X.; Zhou, L. Beauvericin from the endophytic fungus, *Fusarium redolens*, isolated from *Dioscorea zingiberensis* and its antibacterial activity. *Nat. Prod. Commun.* **2010**, *5*, 1934578X1000500527.

83. Meca, G.; Sospedra, I.; Soriano, J.M.; Ritieni, A.; Moretti, A.; Manes, J. Antibacterial effect of the bioactive compound beauvericin produced by *Fusarium proliferatum* on solid medium of wheat. *Toxicon* **2010**, *56*, 349-354, doi:10.1016/j.toxicon.2010.03.022.
84. Jiang, Z.; Barret, M.-O.; Boyd, K.G.; Adams, D.R.; Boyd, A.S.; Burgess, J.G. JM47, a cyclic tetrapeptide HC-toxin analogue from a marine *Fusarium* species. *Phytochemistry* **2002**, *60*, 33-38, doi:10.1016/s0031-9422(02)00061-4.
85. Meca, G.; Sospedra, I.; Valero, M.A.; Manes, J.; Font, G.; Ruiz, M.J. Antibacterial activity of the enniatin B, produced by *Fusarium tricinctum* in liquid culture, and cytotoxic effects on Caco-2 cells. *Toxicol. Mech. Method.* **2011**, *21*, 503-512, doi:10.3109/15376516.2011.556202.
86. Firakova, S.; Šturdíková, M.; Liptaj, T.; Prónayová, N.; Bezáková, L.; Proksa, B. Enniatins produced by *Fusarium dimerum*, an endophytic fungal strain. *Pharmazie* **2008**, *63*, 539-541.
87. Tsantrizos, Y.S.; Xu, X.-J.; Sauriol, F.; Hynes, R.C. Novel quinazolinones and enniatins from *Fusarium lateritium* Nees. *Can. J. Chem.* **1993**, *71*, 1362-1367, doi:10.1139/v93-176.
88. Hawas, U.W.; Al-Farawati, R.; Abou El-Kassem, L.T.; Turki, A.J. Different culture metabolites of the Red Sea fungus *Fusarium equiseti* optimize the inhibition of Hepatitis C Virus NS3/4A Protease (HCV PR). *Mar. Drugs* **2016**, *14*, 190-201, doi:10.3390/md14100190.
89. Chang, S.; Yan, B.; Chen, Y.; Zhao, W.; Gao, R.; Li, Y.; Yu, L.; Xie, Y.; Si, S.; Chen, M. Cytotoxic hexadepsipeptides and anti-coronaviral 4-hydroxy-2-pyridones from an endophytic *Fusarium* sp. *Front. Chem.* **2022**, *10*, 1106869-1106877, doi:10.3389/fchem.2022.1106869.
90. McKee, T.C.; Bokesch, H.R.; McCormick, J.L.; Rashid, M.A.; Spielvogel, D.; Gustafson, K.R.; Alavanja, M.M.; Cardelline, J.H., 2nd; Boyd, M.R. Isolation and characterization of new anti-HIV and cytotoxic leads from plants, marine, and microbial organisms. *J. Nat. Prod.* **1997**, *60*, 431-438, doi:10.1021/np970031g.
91. Guo, Y.W.; Liu, X.J.; Yuan, J.; Li, H.J.; Mahmud, T.; Hong, M.J.; Yu, J.C.; Lan, W.J. L-Tryptophan induces a marine-derived *Fusarium* sp. to produce indole alkaloids with activity against the Zika Virus. *J. Nat. Prod.* **2020**, *83*, 3372-3380, doi:10.1021/acs.jnatprod.0c00717.
92. Nonaka, K.; Chiba, T.; Suga, T.; Asami, Y.; Iwatsuki, M.; Masuma, R.; Ōmura, S.; Shiomi, K. Coculnol, a new penicillic acid produced by a coculture of *Fusarium solani* FKI-6853 and *Talaromyces* sp. FKA-65. *J. Antibiot (Tokyo).* **2015**, *68*, 530-532, doi:10.1038/ja.2015.15.
93. A. Abdelhakim, I.; Bin Mahmud, F.; Motoyama, T.; Futamura, Y.; Takahashi, S.; Osada, H. Dihydrolucilactaene, a potent antimalarial compound from *Fusarium* sp. RK97-94. *J. Nat. Prod.* **2021**, *85*, 63-69, doi:10.1021/acs.jnatprod.1c00677.
94. Balan, J.; Fuska, J.; Kuhr, I.; Kuhrová, V. Bikaverin, an antibiotic from *Gibberella fujikuroi*, effective against *Leishmania brasiliensis*. *Folia Microbiol.* **1970**, *15*, 479-484, doi:10.1007/bf02880192.
95. Campos, F.F.; Sales Junior, P.A.; Romanha, A.J.; Araújo, M.S.; Siqueira, E.P.; Resende, J.M.; Alves, T.; Martins-Filho, O.A.; Santos, V.L.d.; Rosa, C.A. Bioactive endophytic fungi isolated from *Caesalpinia echinata* Lam.(Brazilwood) and identification of beauvericin as a trypanocidal metabolite from *Fusarium* sp. *Mem. Inst. Oswaldo Cruz* **2015**, *110*, 65-74, doi:10.1590/0074-02760140243.
96. Nascimento, A.M.d.; Conti, R.; Turatti, I.C.; Cavalcanti, B.C.; Costa-Lotufo, L.V.; Pessoa, C.; Moraes, M.O.d.; Manfrim, V.; Toledo, J.S.; Cruz, A.K. Bioactive extracts and chemical

constituents of two endophytic strains of *Fusarium oxysporum*. *Rev. Bras. Farmacogn.* **2012**, *22*, 1276-1281, doi:10.1590/s0102-695x2012005000106.

97. Tadpetch, K.; Chukong, C.; Jeanmard, L.; Thiraporn, A.; Rukachaisirikul, V.; Phongpaichit, S.; Sakayaroj, J. Cytotoxic naphthoquinone and a new succinate ester from the soil fungus *Fusarium solani* PSU-RSPG227. *Phytochem. Lett.* **2015**, *11*, 106-110, doi:10.1016/j.phytol.2014.11.018.

98. Ibrahim, S.R.; Mohamed, G.A.; Ross, S.A. Integracides F and G: New tetracyclic triterpenoids from the endophytic fungus *Fusarium* sp. *Phytochem. Lett.* **2016**, *15*, 125-130, doi:10.1016/j.phytol.2015.12.010.

99. Ibrahim, S.R.; Abdallah, H.M.; Mohamed, G.A.; Ross, S.A. Integracides HJ: New tetracyclic triterpenoids from the endophytic fungus *Fusarium* sp. *Fitoterapia* **2016**, *112*, 161-167, doi:10.1016/j.fitote.2016.06.002.

100. Kato, S.; Motoyama, T.; Futamura, Y.; Uramoto, M.; Nogawa, T.; Hayashi, T.; Hirota, H.; Tanaka, A.; Takahashi-Ando, N.; Kamakura, T. Biosynthetic gene cluster identification and biological activity of lucilactaene from *Fusarium* sp. RK97-94. *Biosci., Biotechnol., Biochem.* **2020**, *84*, 1303-1307, doi:10.1080/09168451.2020.1725419.

101. Abdelhakim, I.A.; Motoyama, T.; Nogawa, T.; Mahmud, F.B.; Futamura, Y.; Takahashi, S.; Osada, H. Isolation of new lucilactaene derivatives from P450 monooxygenase and aldehyde dehydrogenase knockout *Fusarium* sp. RK97-94 strains and their biological activities. *J. Antibiot (Tokyo).* **2022**, *75*, 361-374, doi:10.1038/s41429-022-00529-3.

102. Singh, S.B.; Zink, D.L.; Polishook, J.D.; Dombrowski, A.W.; Darkin-Rattray, S.J.; Schmatz, D.M.; Goetz, M.A. Apicidins: Novel cyclic tetrapeptides as coccidiostats and antimalarial agents from *Fusarium pallidoroseum*. *Tetrahedron Lett.* **1996**, *37*, 8077-8080, doi:10.1016/0040-4039(96)01844-8.

103. Singh, S.B.; Zink, D.L.; Liesch, J.M.; Dombrowski, A.W.; Darkin-Rattray, S.J.; Schmatz, D.M.; Goetz, M.A. Structure, histone deacetylase, and antiprotozoal activities of apicidins B and C, congeners of apicidin with proline and valine substitutions. *Org. Lett.* **2001**, *3*, 2815-2818, doi:10.1021/ol016240g.

104. Niehaus, E.-M.; Janevska, S.; von Bargen, K.W.; Sieber, C.M.; Harrer, H.; Humpf, H.-U.; Tudzynski, B. Apicidin F: characterization and genetic manipulation of a new secondary metabolite gene cluster in the rice pathogen *Fusarium fujikuroi*. *PLoS one* **2014**, *9*, e103336, doi:10.1371/journal.pone.0103336.

105. Von Bargen, K.W.; Niehaus, E.-M.; Bergander, K.; Brun, R.; Tudzynski, B.; Humpf, H.-U. Structure elucidation and antimalarial activity of apicidin F: an apicidin-like compound produced by *Fusarium fujikuroi*. *J. Nat. Prod.* **2013**, *76*, 2136-2140, doi:10.1021/np4006053.
